# A prognostic 19-gene signature and LBP-mediated immune dysregulation define the tumor microenvironment in poor-prognosis KIRC

**DOI:** 10.7150/ijms.125505

**Published:** 2026-01-30

**Authors:** Chia-Hung Chen, Hsiao-Hsuan Huang, Tzu-Han Weng, Ta-Wei Kuo, Nien-Che Ho, Kai-Yao Huang, Hui-Ju Kao, Chen-Lin Yu, Shun-Long Weng, Kuang-Wen Liao

**Affiliations:** 1 Department of Medical Research, Hsinchu MacKay Memorial Hospital, Hsinchu City 30071, Taiwan, ROC.; 2 Department of Medical Research, Hsinchu Municipal MacKay Children's Hospital, Hsinchu City 30068, Taiwan, ROC.; 3 Industrial Development Graduate Program of College of Engineering Bioscience, National Yang Ming Chiao Tung University, Hsinchu City 30068, Taiwan, ROC.; 4 Department of Dermatology, MacKay Memorial Hospital, Taipei City 10449, Taiwan, ROC.; 5 Department of Biological Science and Technology, College of Engineering Bioscience, National Yang Ming Chiao Tung University, Hsinchu City 30068, Taiwan, ROC.; 6 Department of Medicine, MacKay Medical College, New Taipei City 25245, Taiwan, ROC.; 7 Institute of Biomedical Sciences, MacKay Medical College, New Taipei City 25245, Taiwan, ROC.; 8 Department of Obstetrics and Gynecology, Hsinchu MacKay Memorial Hospital, Hsinchu City 30071, Taiwan, ROC.; 9 Mackay Junior College of Medicine, Nursing and Management, Taipei City 11260, Taiwan, ROC.; 10 Department of Obstetrics and Gynecology, Hsinchu Municipal MacKay Children's Hospital, Hsinchu City 30068, Taiwan, ROC.; 11 Institute of Molecular Medicine and Bioengineering, College of Engineering Bioscience, National Yang Ming Chiao Tung University, Hsinchu City 30068, Taiwan, ROC.; 12 Center for Intelligent Drug Systems and Smart Bio-devices (IDS 2 B), National Yang Ming Chiao Tung University, Hsinchu City 30068, Taiwan, ROC.; 13 Department of Biotechnology and Bioindustry Sciences, National Cheng Kung University, Tainan City 70101, Taiwan, ROC.; 14 Graduate Institute of Medicine, College of Medicine, Kaohsiung Medical University, Kaohsiung City 80708, Taiwan, ROC.; 15 School of Dentistry, Kaohsiung Medical University, Kaohsiung City 80708, Taiwan, ROC.; 16 Drug Development and Value Creation Research Center, Kaohsiung Medical University, Kaohsiung City 80708, Taiwan, ROC.

**Keywords:** kidney renal clear cell carcinoma (KIRC), tumor microenvironment (TME), lipopolysaccharide-binding protein (LBP), prognostic gene signature

## Abstract

Kidney renal clear cell carcinoma (KIRC) exhibits pronounced immune heterogeneity, and immune dysregulation within the tumor microenvironment (TME) contributes to poor outcomes. Leveraging TCGA-KIRC RNA-seq, we stratified patients by immune-cell infiltration and immune-regulatory gene expression to define a poor-survival subgroup for discovery. Differential expression analysis prioritized lipopolysaccharide-binding protein (LBP) and generated an immune-relevant candidate set that was refined from 406 to 87 genes by stepwise logistic regression and then benchmarked through one million random 20-gene models, yielding a final 19-gene prognostic signature. Six immune-cell features associated with survival were identified, including higher M0 macrophages, regulatory T cells, activated CD4 memory T cells, plasma cells, and neutrophils (worse prognosis) and resting mast cells (better prognosis). LBP was highly expressed in the poor-survival subgroup and functionally validated *in vitro*: RT-PCR/ELISA/Western blot and cell-based assays showed that LBP promotes tumor-cell migration and macrophage activation, while LBP neutralization reversed these effects, supporting its role as a mediator of tumor-immune crosstalk. The 19-gene panel robustly distinguished poor-survival patients, achieving AUC = 0.84 in TCGA-KIRC and 0.79-1.00 across three external datasets. Pathway analysis implicated ERK signaling, immune suppression, and chronic inflammation. These findings establish a clinically relevant 19-gene signature and highlight LBP-driven immune dysregulation as a potential target in KIRC.

## Introduction

Kidney renal clear cell carcinoma (KIRC), the most common subtype of renal cell carcinoma, accounts for approximately 70-80% of all cases. Although the advent of targeted therapies and immune checkpoint inhibitors (ICIs) has improved outcomes in a subset of patients, those with advanced-stage KIRC continue to experience poor prognoses 1-3. Despite being considered a highly immunogenic tumor, KIRC exhibits substantial heterogeneity in immune cell infiltration and therapeutic response 4, 5. Some tumors resemble “cold” tumors with minimal immune cell presence, while others show extensive immune infiltration yet paradoxically associate with unfavorable survival outcomes 6. These findings highlight that immune contexture—the composition, functional status, and spatial organization of immune cells—is more informative than mere immune cell abundance when assessing prognosis and treatment response 7, 8. This complexity reflects the broader regulatory role of the tumor microenvironment (TME), which orchestrates tumor development, immune evasion, and resistance to therapy through intricate crosstalk between tumor, immune, and stromal components 9, 10. Traditionally, tumors are categorized as “hot” or “cold” based on immune cell infiltration levels, with hot tumors typically responding better to immunotherapy, whereas cold tumors tend to be resistant 11-14. However, immune infiltration alone does not guarantee favorable outcomes. In certain cancers, increased immune cell presence has paradoxically correlated with worse survival, further emphasizing that the quality and function of immune components—not just their quantity—are critical determinants of clinical outcomes 15. Therefore, a deeper understanding of immune dynamics within the KIRC microenvironment is urgently needed to identify robust prognostic biomarkers and to guide the development of more effective, personalized immunotherapeutic strategies 6, 16.

In this study, we aimed to identify immune-related molecular features associated with poor survival in KIRC and to investigate their potential mechanistic relevance. Using bulk RNA-seq data from TCGA-KIRC, we performed dual patient stratification based on immune cell composition (via CIBERSORTx) and immune regulatory gene expression (via Gene Ontology analysis). We then focused on patients classified into poor-prognosis groups by both methods to define a robust cohort for downstream analysis. Through systematic gene prioritization and statistical refinement, we constructed a 19-gene panel enriched for immune regulatory genes consistently associated with adverse outcomes. Cross-dataset validation and functional annotation revealed that these genes are involved in key processes, including immune suppression, inflammation, and tumor invasion.

Among these, lipopolysaccharide-binding protein (LBP) emerged as a prominent candidate due to its high expression in tumors with poor survival rates and its centrality in the network of tumor and immune pathways. While traditionally known for its role in innate immunity, recent studies have linked LBP to tumor-promoting inflammation and immune modulation 17-19. To explore its functional relevance in the KIRC microenvironment, we further investigated LBP's effects using *in vitro* models of tumor-immune interaction.

## Materials and Methods

### RNA sequencing and microarray datasets

RNA sequencing, gene expression microarray and clinical data were obtained from four public datasets: TCGA-KIRC, GSE22541, E-MTAB-1980, and RECA-EU. All expression data were processed using R (v3.6.2).

RNA sequencing (RNA-seq) data and corresponding clinical information for KIRC were retrieved from The Cancer Genome Atlas (TCGA) database using the *TCGAbiolinks* package in R. Gene expression levels were normalized and reported as fragments per kilobase of transcript per million mapped reads (FPKM). A total of 539 KIRC patient samples were included in the analysis. To ensure consistent gene annotation, the expression matrix was mapped to Ensembl gene identifiers (ENSG) and gene symbols according to the GRCh38/hg38 human reference genome. For downstream analyses, only protein-coding genes were retained to improve biological interpretability and reduce potential confounding from non-coding RNAs.

The GSE22541 and E-MTAB-1980 microarray datasets were downloaded from GEO and ArrayExpress, respectively; preprocessing details follow repository documentation 20. RNA-seq data from the RECA-EU cohort were obtained from the ICGC portal and include European patients with renal cancer.

### Annotation of immune regulation genes using the Gene Ontology database

Genes involved in immune regulation were identified using the Gene Ontology (GO) resource 21, 22. The search term “immune regulation” was applied under the “Ontology” subclass, and genes annotated with the accession ID GO:0050776 (regulation of immune response) were selected. These genes were filtered from the previously curated protein-coding gene list, resulting in a final set of 987 immune regulation genes for further analysis.

### CIBERSORTx analysis for immune cell quantification

Cell-type Identification by Estimating Relative Subsets of RNA Transcripts (CIBERSORTx) is an online computational tool designed to quantify immune cell composition based on gene expression data 23. CIBERSORTx utilizes a reference gene matrix consisting of 547 signature genes to deconvolute bulk RNA-sequencing data into the relative proportions of 22 immune cell types. This approach allowed us to determine the immune cell content in the TME of renal cancer patients by applying CIBERSORTx's deconvolution algorithm to the mRNA expression profiles.

### Gaussian Mixture Model (GMM) clustering

Gaussian Mixture Modeling (GMM) was applied as an unsupervised clustering approach to identify immune infiltration patterns across tumor samples. GMM assumes that the observed data arise from a mixture of multiple Gaussian distributions and is well suited for modeling heterogeneous and multimodal data structures commonly observed in immune deconvolution profiles. Unlike hard clustering methods, GMM provides probabilistic cluster assignments, allowing samples to exhibit gradual transitions between immune states rather than being forced into rigid cluster boundaries.

In this study, samples were represented in a high-dimensional feature space defined by immune cell composition profiles and clustered using the “mclust” package in R (version 3.6.2). This approach enabled the identification of distinct immune infiltration patterns across the dataset.

### Kaplan-Meier survival analysis

Kaplan-Meier (KM) survival analysis was performed to evaluate the overall survival (OS) of patients within distinct clusters or immune cell groups. For GMM-based clustering, patient groups were analyzed using the “survival” and “survminer” packages in R.

To stratify KIRC patients based on immune landscape, the cohort was classified into high and low immune cell infiltration groups according to the variability in infiltration levels across 22 distinct immune cell types. This stratification approach is particularly suited for data characterized by a long-tailed distribution and aims to partition the samples into two different subgroups. The procedure involves initially ranking all samples based on the variable of interest. Subsequently, the standard deviation (SD) is sequentially calculated in an accumulative manner—from the first three samples, then the first four, and so forth. Since the standard deviation reflects the degree of dispersion within a potential cluster, this iterative process allows the construction of a simulation curve. The point at which the SD reaches its maximum is identified as the optimal cut-off, serving as the threshold to divide the cohort into two subgroups: one with relatively high infiltration values and the other with low infiltration values.

Clinical data and group labels were integrated into the analysis, and survival curves were generated. Statistical significance was assessed using the log-rank test, with a threshold of p < 0.05 to define significant survival differences.

### Stepwise logistic regression algorithm

In this study, the expression levels of immune regulatory genes served as independent variables, while patient survival status (alive vs. dead) was used as the binary response variable. The logistic regression model estimates the probability of the outcome using a logistic function, with model coefficients derived through Maximum Likelihood Estimation (MLE). To identify the most informative subset of genes associated with poor survival, we applied a stepwise selection algorithm that combines both forward selection and backward elimination. The procedure was initialized with a null model and iteratively added or removed variables based on their contribution to model fit. Selection criteria were based on either the Akaike Information Criterion (AIC) or variable-level p-values, and the process continued until no further improvement could be achieved by modifying the model. The stepwise regression was implemented in R (version 3.6.2) using the built-in step() function. The resulting gene subsets were used for downstream ROC analysis, frequency profiling, and prognostic panel construction. This strategy allowed functional diversity to be retained while quantitatively optimizing prognostic performance.

### Receiver operating characteristic (ROC) curve analysis

For single-gene evaluation, patients were stratified into binary survival groups, and the ROC curve was generated using the R package pROC (version 1.18.0). For multi-gene combinations, a logistic regression model was constructed, and predicted probabilities were used to compute the ROC curve. In comparisons involving multiple models or gene sets, we generated AUC distributions and visualized them using ggplot2 (version 3.3.5) to assess relative performance.

Where applicable, the DeLong test was used to compare AUCs between different classifiers. All analyses were conducted using R software (version 3.6.2), and statistical significance was defined as *p* < 0.05.

### RT-PCR

786-O or THP-1 cells (1 × 10^6^ cells/well) were treated with LPS (5 ng/mL) for 12 hours. Total RNA was extracted using TRIzol reagent (Invitrogen, Gaithersburg, MD, USA) following the manufacturer's instructions. The extracted RNA was then reverse transcribed into cDNA. To detect gene expression, specific primers targeting LBP and β-actin were used. The LBP gene was amplified using a forward primer with the sequence 5′-TTCGGTCAACCTCCTGTTGG-3′ and a reverse primer 5′-CATGTTGGGGTAGAGCCTGG-3′. β-actin was used as the internal control, with the forward primer 5′-CACCATTGGCAATGAGCGGTTC-3′ and the reverse primer 5′-AGGTCTTTGCGGATGTCCACGT-3′. PCR products were separated by agarose gel electrophoresis.

### Wound healing assay

786-O cells (3 × 10⁴ cells/70 μL per well) were seeded into a Culture-Insert 2 Well (Ibidi, Munich, Germany) and incubated overnight to allow cell attachment. The inserts were then carefully removed to create a uniform cell-free gap. Cell migration into the gap was monitored at 0 and 24 hours using an inverted microscope, and representative images were captured at each time point. Migration was quantified by measuring the change in the gap width between the two cell fronts over time. The migration rate was calculated as: [(initial gap width - final gap width) / initial gap width] × 100.

### ELISA

The concentration of LBP in cell culture supernatants was measured using commercially available ELISA kits (Invitrogen). Briefly, culture media were collected, centrifuged to remove debris, and added to pre-coated 96-well plates. After incubation and washing, detection antibodies were added, followed by substrate solution. Absorbance was measured at 450 nm using a microplate reader, and protein concentrations were calculated based on standard curves.

### Statistical analysis

All data are presented as mean ± standard deviation (SD) from at least three independent experiments. For comparisons between two groups, either Student's *t*-test or the Wilcoxon rank-sum test was applied based on the normality of the data distribution. For comparisons involving more than two groups, one-way analysis of variance (ANOVA) was performed, followed by appropriate post-hoc tests to assess pairwise group differences. A *p*-value of < 0.05 was considered statistically significant.

## Results

### Immune infiltration landscape in KIRC and its prognostic implications

To explore the heterogeneity of the TME in KIRC, we employed CIBERSORTx to quantify 22 immune cell types from the RNA-seq data of TCGA-KIRC samples. Unsupervised clustering using a GMM classified patients into five distinct immune infiltration groups (Groups A-E), each displaying specific immune cell enrichment patterns (Fig. S1A). For example, Group A showed high levels of resting NK cells, Group C was enriched in gamma delta T cells, and Groups D and E had prominent activated mast cells and M0 macrophages, respectively. Group B, the largest cluster, lacked dominance by a specific immune cell.

**Figure 1:**
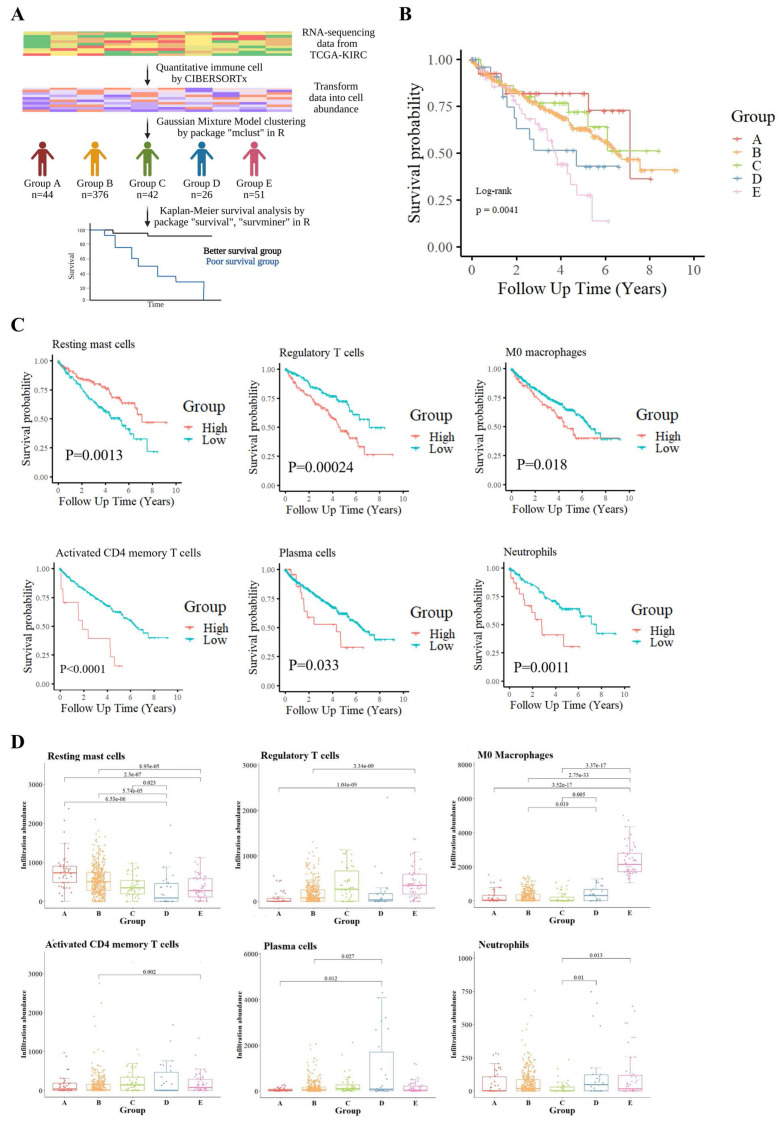
** Immune cell-based classification and its association with survival in the TCGA-KIRC cohort. (A)** Analysis workflow for patient stratification based on tumor immune cell composition. Relative proportions of 22 immune cell types were estimated using the CIBERSORTx algorithm applied to TCGA-KIRC RNA-seq data. Patients were clustered into five immune subgroups (Groups A-E) using GMM, and overall survival was compared across groups. **(B)** Kaplan-Meier survival curves showing significant survival differences among the five immune subgroups (log-rank p = 0.0041). **(C)** Kaplan-Meier analysis identifying six immune cell types significantly associated with prognosis. Elevated levels of regulatory T cells, M0 macrophages, activated CD4⁺ memory T cells, plasma cells, resting mast cells, and neutrophils were each linked to shorter survival. **(D)** Boxplots illustrate the distribution of these six prognostic immune cell types across the five subgroups. Statistical comparisons were conducted using the Wilcoxon test, with p-values shown.

KM survival analysis was conducted to assess the relationship between immune cell infiltration patterns and patient prognosis across the five clusters (Fig. 1B). Results demonstrated that patients in Groups D and E had significantly poorer outcomes, indicating a strong association between immune cell infiltration patterns and survival. To explore this further, the 22 immune cell types were stratified into high-infiltration and low-infiltration groups based on the variability in infiltration. Survival comparisons between these groups identified six immune cell types significantly associated with patient survival (Fig. 1C). Resting mast cell infiltration was positively correlated with better survival outcomes, while five other cell types—including M0 macrophages, regulatory T cells (Tregs), plasma cells, activated CD4 memory T cells, and neutrophils—were negatively correlated. Additionally, naïve CD4 T cells were excluded from statistical analysis due to insufficient data, while the remaining 15 immune cell types showed no significant association with survival (Fig. S1B).

Boxplot comparisons of these immune cell infiltrates across the five immune clusters, accompanied by Wilcoxon tests, revealed distinct distribution patterns, further emphasizing their prognostic relevance (Fig. 1D). Notably, M0 macrophages were most abundant in Groups D and E, linking them to tumor aggressiveness and poor survival outcomes.

### Defining a poor-survival KIRC patient group for molecular interrogation

**Figure 2:**
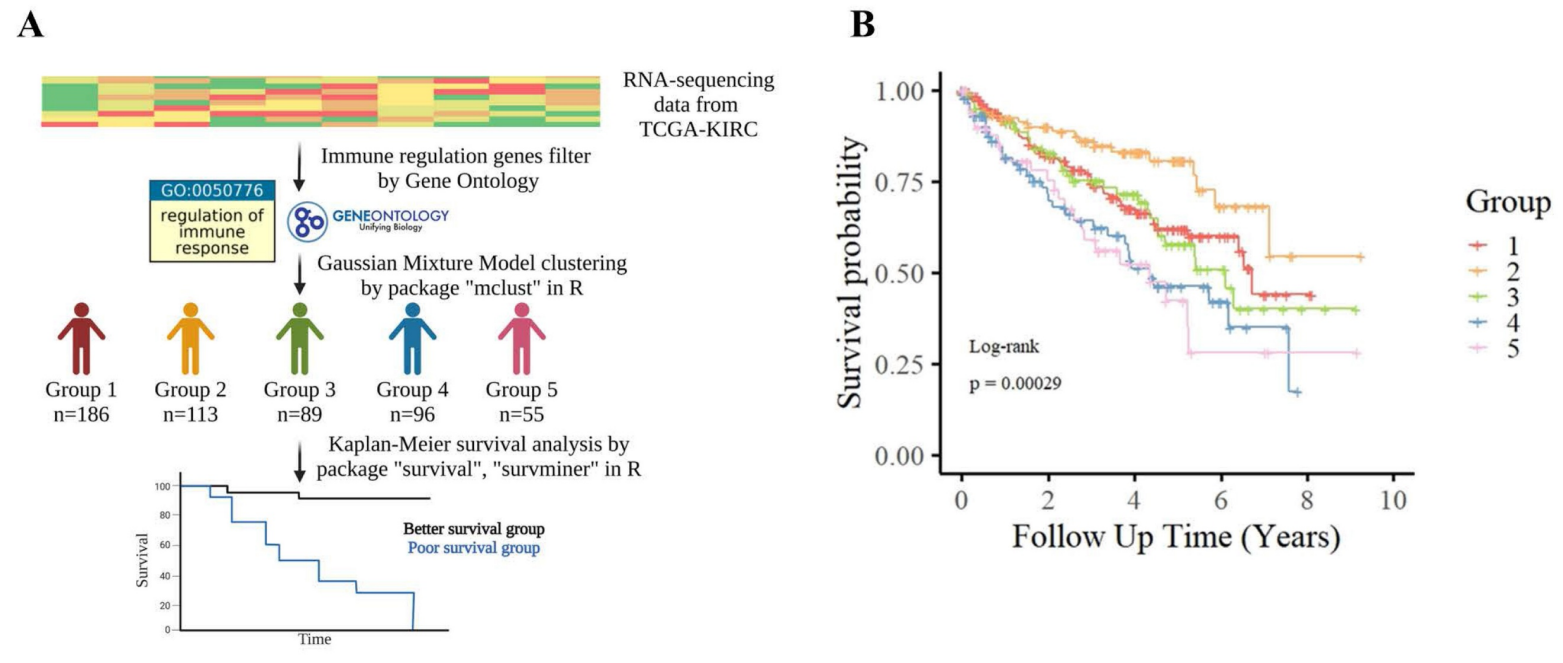
** Patient stratification based on immune regulation-related gene expression in TCGA-KIRC. (A)** TCGA-KIRC RNA-sequencing data were filtered for genes associated with the Gene Ontology term “regulation of immune response” (GO:0050776). Patients were clustered into five transcriptomic subgroups (Groups 1-5) using GMM based on the expression patterns of these immune regulation-related genes. **(B)** Kaplan-Meier survival analysis showed significant differences in overall survival among the five subgroups (log-rank p = 0.00029), suggesting that variation in immune regulatory gene expression is closely linked to patient prognosis in KIRC.

In parallel with immune profiling, we performed transcriptomic clustering based on immune regulatory gene expression—defined by GO term GO:0050776—and identified five molecular subgroups (Groups 1-5) via GMM (Fig. 2A). KM analysis showed that Groups 4 and 5 had significantly poorer prognosis (Fig. 2B). We intersected this classification with the immune infiltration analysis, identifying 38 overlapping patients from immune Groups D/E and transcriptomic Groups 4/5, which we collectively defined as the poor-survival group.

For tumor samples in this group, an immune gene index was calculated by multiplying each gene's expression value by the fold change (FC) between tumor and adjacent normal tissues. Based on the calculated index, genes were ranked in descending order. The top 20 candidates are presented in Table 1, along with their detailed functional annotations, also listed in Table 1. Additional gene data are available in Supplementary Table S1. Notably, a significant drop in index values was observed after the fifth-ranked gene, leading to the selection of the top five candidates—*LBP*, *COL1A1*, *FGA*, *FGB*, and *C3*—for further analysis.

**Figure 3:**
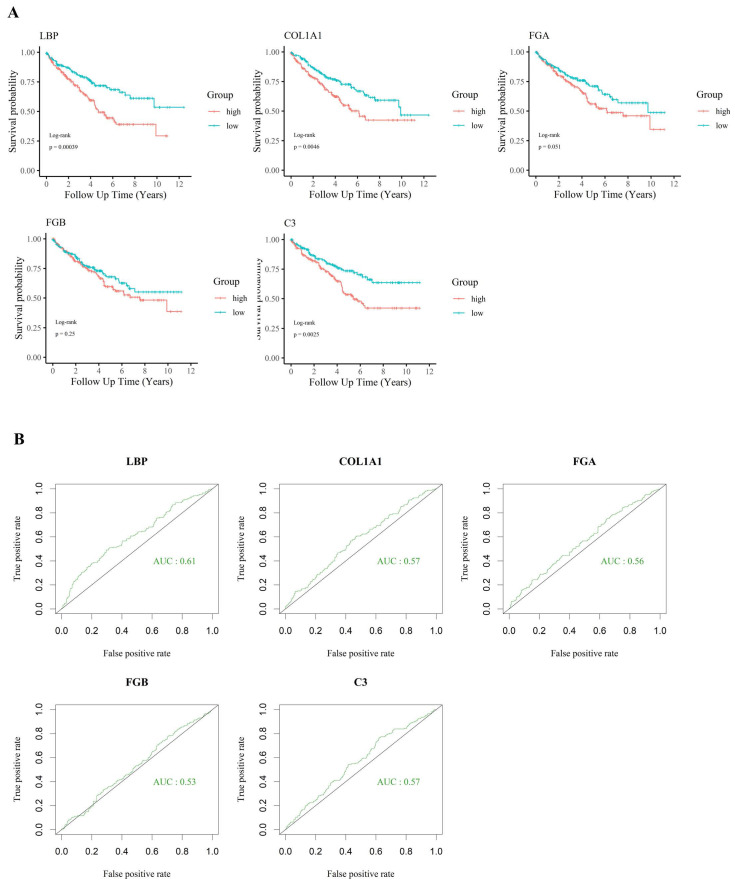
** Prognostic significance of selected immune-related genes in KIRC. (A)** Kaplan-Meier survival analysis of five candidate genes (*LBP*, *COL1A1*, *FGA*, *FGB*, and *C3*) in the TCGA-KIRC cohort. High expression of LBP, COL1A1, and C3 was significantly associated with worse overall survival (p < 0.05), suggesting their involvement in poor prognosis. **(B)** ROC curve analysis showing the relative ability of each gene to distinguish between survival outcomes. LBP had the highest AUC (0.61), while the others had AUC values below 0.6, supporting the prioritization of LBP for further investigation despite modest standalone performance.

KIRC patients were further stratified into high-expression (top one-third) and low-expression (bottom one-third) groups based on the expression levels of the five candidate genes. Survival analysis (Fig. 3A) revealed that the expression of *LBP*, *COL1A1*, and *C3* was significantly associated with patient outcomes. Among these genes, *LBP* had the highest AUC (> 0.6) in ROC analysis (Fig. 3B), suggesting a closer relationship with adverse clinical outcomes and highlighting its potential biological relevance in poor-prognosis KIRC. Based on the immune gene index, survival correlation, and ROC performance, *LBP* was identified as the most influential gene and selected as the key effector for further investigation. Subsequently, a cohort of 38 poor-survival KIRC patients exhibiting high LBP expression was defined, providing a focused population for downstream immunogenomic characterization and molecular analysis.

### Identification of core immunoregulatory genes associated with poor survival in high-LBP KIRC patients

**Figure 4:**
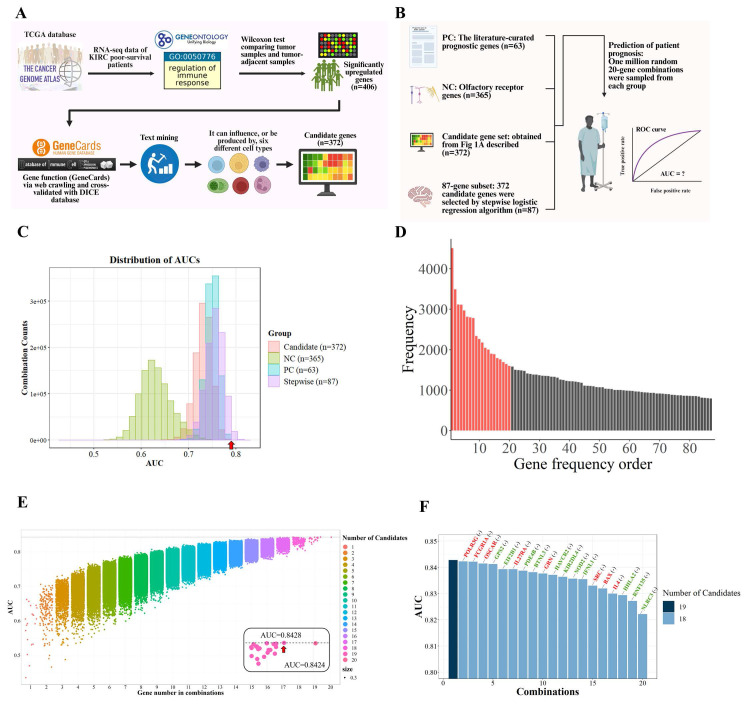
** Systematic identification and refinement of immune-related genes associated with poor survival in KIRC. (A)** Flowchart summarizing the pipeline. Differentially expressed immune-regulatory genes associated with poor survival were mapped to six immune cell types, yielding 372 candidates refined by stepwise logistic regression. **(B)** Prognostic relevance was compared across four sets: (1) PC: 63 literature-curated genes, (2) NC: 365 olfactory receptor genes, (3) 372 immune-related candidates, and (4) 87 stepwise-selected genes. One million random 20-gene combinations from each group were tested for survival prediction. **(C)** AUC distributions showed the 87-gene subset consistently outperformed others. The PC benchmark (AUC = 0.797, red arrow) was used as a threshold. **(D)** Genes were ranked by frequency in combinations exceeding the benchmark, identifying 20 top candidates (red). **(E)** Exhaustive modeling revealed a 19-gene panel achieved optimal performance (AUC = 0.8428). **(F)** Leave-one-gene-out analysis showed that removing any gene reduced performance, indicating synergy. *NLRC3*, *RNF135*, *HHLA2*, *IL4*, *BAX*, and *SRC* had the strongest effects. Red genes are upregulated in poor-survival patients, whereas green genes are downregulated.

Given the high expression of LBP—a key acute-phase protein involved in immune responses—in poor-prognosis KIRC patients, we aimed to characterize the associated immunoregulatory landscape and identify co-expressed genes that may contribute to both tumor aggressiveness and immune suppression. To this end, we focused on tumor tissues from the same 38 poor-survival patients and performed differential expression analysis compared to their matched adjacent normal tissues. This analysis identified 406 upregulated genes (Wilcoxon test, p < 0.05; Fig. 4A).

These 406 genes were first functionally annotated using GeneCards and cross-referenced with the DICE database to assess their immune cell-specific expression profiles. To enrich for immunologically relevant candidates, we filtered for genes expressed in six immune cell types previously linked to poor survival in KIRC—M0 macrophages, Tregs, neutrophils, plasma cells, activated CD4⁺ memory T cells, and resting mast cells. This yielded a refined list of 372 genes with potential immunoregulatory roles in tumor progression, which served as the foundation for subsequent modeling and analysis.

To further refine genes with the highest statistical association with patient outcomes, stepwise logistic regression was employed on this 372-gene set, resulting in a focused panel of 87 candidates. These genes served as the basis for downstream functional prioritization and mechanistic investigation.

To identify which of the 87 immune-related candidates most significantly contribute to poor survival in KIRC, a large-scale benchmarking analysis was performed. We generated one million random 20-gene combinations from the 87-gene subset and evaluated their survival-predictive performance using logistic regression and AUC analysis. These combinations were compared against two control sets, detailed in Table S2: a positive control (PC) of 63 literature-curated prognostic genes 24, 25, and a negative control (NC) of 365 olfactory receptor genes unlikely to be associated with cancer outcomes (Fig. 4B). Although the performance of candidate gene combinations was primarily evaluated using AUC-based benchmarking, functional complementarity among genes was implicitly preserved through the upstream filtering strategy. Prior to random modeling, candidate genes were restricted to those that were (i) significantly upregulated in tumors from poor-survival patients, (ii) annotated as immune-regulatory genes (GO:0050776), and (iii) expressed in immune cell populations that were significantly associated with survival. This multi-layered filtering ensured that the resulting gene pool represented coordinated immune suppression, inflammatory signaling, antigen presentation, and tumor-intrinsic survival pathways, rather than redundant predictors driven by single mechanisms.

The distribution of AUC scores (Fig. 4C) showed that gene combinations from our 87-gene subset consistently outperformed the PC group and far exceeded the NC baseline, underscoring the robustness of our biologically informed and statistically refined gene selection strategy. These benchmarking results served to quantitatively highlight genes most strongly associated with poor survival outcomes.

Based on this analysis, we identified the top 20 most frequently occurring genes among high-performing combinations (AUC > 0.797), suggesting these genes may be central contributors to the immunopathology of aggressive KIRC (Fig. 4D and Table S3). Exhaustive testing of all possible combinations within this 20-gene set revealed that a 19-gene panel achieved the highest association with poor survival (AUC = 0.8428; Fig. 4E). To evaluate the contribution of individual genes to model performance, we conducted a leave-one-gene-out analysis. Notably, the exclusion of genes such as *NLRC3*, *RNF135*, *HLA-A2*, *IL4*, *BAX*, and *SRC* led to marked declines in AUC, highlighting their critical contribution to the overall performance of the gene panel and supporting their biological relevance (Fig. 4F). The remaining genes contributed synergistically, suggesting that the panel reflects coordinated immunological dysregulation rather than isolated markers.

In summary, the final 19-gene panel was selected to represent the key molecular features underlying immune suppression and tumor aggressiveness in poor-survival KIRC patients. Functional annotations of these genes are provided in Table 2, highlighting their diverse roles in inflammation, immune signaling, and tumor cell regulation.

### Cross-platform validation of immune gene panel relevance

**Figure 5:**
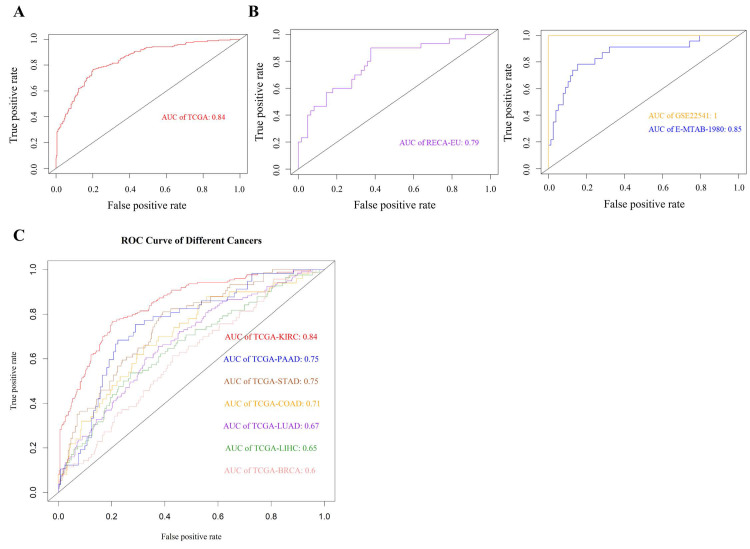
** Cross-dataset validation and cancer-type specificity of the 19-gene panel associated with poor survival in KIRC. (A)** ROC curve showing that the 19-gene panel effectively distinguishes poor-survival cases in the TCGA-KIRC cohort (AUC = 0.84), supporting its biological relevance in this population. **(B)** Validation across three independent cohorts—RECA-EU (RNA-seq, n = 91), GSE22541 (microarray, n = 68), and E-MTAB-1980 (microarray, n = 101)—demonstrated consistent associations with survival, underscoring the panel's robustness across different platforms and populations. **(C)** Evaluation across six additional TCGA cancer types revealed that the panel's expression pattern was selectively informative in metabolically active tumors such as pancreatic (PAAD, AUC = 0.75), gastric (STAD, AUC = 0.75), and colon (COAD, AUC = 0.71) adenocarcinomas, but showed minimal relevance in breast (BRCA), liver (LIHC), and lung (LUAD) cancers (AUC ≤ 0.7), highlighting its specificity to KIRC-associated immunopathology.

To evaluate the consistency of the 19-gene panel across datasets and platforms, we assessed its association with survival in multiple independent cohorts. In the TCGA-KIRC cohort, the panel demonstrated strong discriminatory capacity (AUC = 0.84; Fig. 5A). Its performance was similarly retained in two microarray datasets—GSE22541 (AUC = 1.0) and E-MTAB-1980 (AUC = 0.84)—as well as in the RNA-seq-based RECA-EU dataset (AUC = 0.79; Fig. 5B). These results confirm that the selected genes are consistently and strongly associated with poor survival in KIRC across diverse technical platforms and patient populations.

### Specificity of immune dysregulation in KIRC compared to other cancers

We next investigated whether the 19-gene panel was uniquely associated with KIRC or reflected a more general signature of aggressive cancer phenotypes. To this end, we applied the panel to six other TCGA cancer types. Moderate associations with poor survival were observed in pancreatic (PAAD), gastric (STAD), and colon adenocarcinoma (COAD) (AUC = 0.71-0.75), but not in lung (LUAD), liver (LIHC), or breast cancer (BRCA), where performance dropped to AUC ≤ 0.6 (Fig. 5C). These results indicate that the 19-gene panel retains moderate prognostic relevance in pancreatic, gastric, and colon adenocarcinomas, all of which arise from inflammation-prone tissues and exhibit prominent involvement of innate immune components, particularly macrophages. In contrast, limited performance was observed in other cancer types, highlighting differences in immune contexture across tissues.

### Functional mapping of immune gene activity in poor-survival KIRC

**Figure 6:**
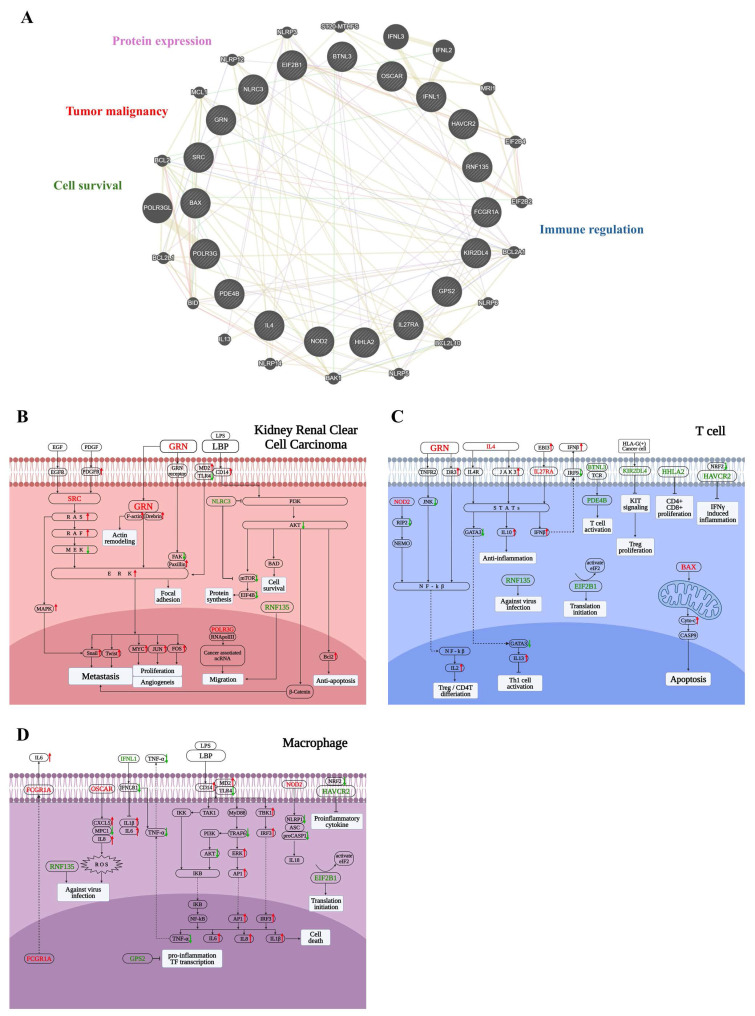
** Functional dissection of the 19-gene panel across tumor cells, T cells, and macrophages. (A)** GeneMANIA network illustrating functional interactions between the 19-gene panel and 20 additional related genes. The network reveals dense connectivity, with functional clusters annotated based on literature, including tumor malignancy (e.g., *GRN*, *SRC*), protein expression (e.g., *EIF2B1*), cell survival and apoptosis (e.g., *BAX*), and immune regulation (e.g., *OSCAR*, *PDE4B*). **(B)** Tumor cell-specific pathway mapping based on KEGG and literature review. Genes such as *SRC*, *GRN*, *RNF135*, and *POLR3G* contribute to epithelial-mesenchymal transition (EMT), invasion, and survival. *LBP* engages the CD14-TLR4 axis, potentially activating inflammatory signaling within tumor cells. Downregulation of *NLRC3* may relieve suppression of mTOR signaling, further enhancing tumor aggressiveness. **(C)** T cell-specific functional diagram showing that *IL4*, *IL27RA*, and *BTNL3* drive Treg/Th2 polarization through JAK-STAT signaling. Reduced expressions of *HAVCR2*, *HHLA2*, and *KIR2DL4* suggests impaired cytotoxic T cell function, while *BAX* upregulation indicates enhanced T cell apoptosis. **(D)** Macrophage-related signaling events indicate activation of NF-κB and inflammasomes by *FCGR1A*, *OSCAR*, and *NOD2*, promoting a chronic inflammatory state. *LBP* enhances these effects by activating the CD14-MD2-TLR4 complex, leading to IRF3 and AP-1 signaling and increased cytokine release. Suppressed expressions of *HAVCR2* and *IFNL1* reflect diminished antiviral and anti-tumor responses, while *GPS2* is involved in transcriptional control of inflammatory genes. In panels (**B**-**D**), red-colored genes represent those significantly upregulated in poor-survival KIRC patients compared to others, while green-colored genes are downregulated in the same comparison. Red arrows indicate pathway activation (upregulation), and green arrows indicate suppression (downregulation).

To better understand the biological significance of the 19-gene panel, we performed network analysis using GeneMANIA (Fig. 6A), revealing tight interconnectivity among genes involved in tumor progression, immune evasion, cell survival, and transcriptional regulation.

To further investigate how these genes may influence the tumor microenvironment in KIRC patients with poor survival, we focused on three key cell types that play pivotal roles in tumor progression: tumor cells, T cells, and macrophages—all of which demonstrated significant associations with patient prognosis. In addition to the 19-gene panel, LBP was also included, which emerged as a top candidate of poor-survival KIRC patients in earlier analyses and exhibited functional relevance across both tumor and immune compartments. To delineate the context-specific functions of these genes within distinct cellular settings, we constructed three cell type-specific molecular pathway diagrams based on KEGG pathway analysis and literature evidence, corresponding to tumor cells, T cells, and macrophages.

To facilitate interpretation of the complex signaling network shown in Figure 6B-6D, key regulatory nodes with opposing functional roles are highlighted below. In particular, imbalanced regulation within major survival pathways, such as PI3K-AKT signaling, provides mechanistic insight into tumor aggressiveness. In tumor cells (Fig. 6B), the 19-gene panel reflected enhanced metastatic and survival potential. Upregulation of *SRC* and *GRN* activated the RAS-RAF-MEK-ERK cascade, promoting cytoskeletal remodeling, focal adhesion, and epithelial-mesenchymal transition (EMT)—hallmarks of metastasis and angiogenesis. *GRN* also enhanced actin remodeling through Drebrin and facilitated ERK-mediated oncogenic transcription programs. *LBP*, via the CD14-MD2-TLR4 complex, activated downstream inflammatory pathways, potentially contributing to tumor-promoting immune modulation. *POLR3G* supported cancer-associated ncRNA transcription, aiding in tumor cell migration. Additionally, *RNF135* and *NLRC3* exhibited opposing roles in PI3K-AKT signaling: *RNF135* promoted survival and anti-apoptosis via β-catenin and Bcl-2, while downregulation of *NLRC3*—an immune checkpoint regulator—facilitated tumor progression. Collectively, these alterations underscore the panel's relevance to tumor aggressiveness and immune evasion in KIRC.

In T cells (Fig. 6C), the 19-gene panel indicated an immunosuppressive and dysfunctional immune state. Upregulation of *IL4*, *IL27RA*, and *BTNL3* promoted JAK-STAT signaling and Treg or Th2 differentiation, thereby dampening anti-tumor immunity. *GRN* and *EBI3* further activated IL10 and IFN-β signaling, contributing to an immunoregulatory environment. In contrast, downregulation of *HAVCR2*, *HHLA2*, and *KIR2DL4*—co-inhibitory and co-stimulatory molecules—suggested impaired CD8⁺ T cell proliferation and effector function. Notably, increased expression of pro-apoptotic *BAX* pointed to elevated T cell apoptosis. Meanwhile, *EIF2B1* and *PDE4B* were involved in translation initiation and cytokine regulation, respectively, though their expression changes were insufficient to restore T cell function. *RNF135* appeared to play a role in antiviral defense, while downregulation of *NOD2* suppressed NF-κB activation and IL-2 production, potentially limiting effector T cell differentiation. Altogether, these alterations reflected a suppressed T cell response in poor-survival KIRC.

In macrophages (Fig. 6D), the 19-gene panel reflected a pro-inflammatory transcriptional state characteristic of tumor-promoting chronic inflammation. *FCGR1A* and *OSCAR* stimulated ROS production and cytokine secretion, while *NOD2* activated inflammasome components and IL-18 release, collectively enhancing IL-1β, IL-6, and IL-8 expression. Upregulation of *LBP* further amplified innate immune signaling through the CD14-MD2-TLR4 axis, activating the MyD88-IRF3 and NF-κB pathways. This cascade promoted transcription of multiple pro-inflammatory cytokines and transcription factors, including AP-1 and IRF3. In contrast, the downregulation of *HAVCR2* and *IFNL1* indicated impaired antiviral and anti-tumor responses. Although *GPS2* and *EIF2B1* participated in transcriptional repression and translational control, their expression appeared insufficient to counteract the prevailing inflammatory milieu. These changes suggest a macrophage phenotype skewed toward tumor-supportive inflammation and immune dysregulation in poor-survival KIRC.

Altogether, these 19 genes span tumor-intrinsic and immune-related mechanisms, highlighting their collective role in tumor aggression, immune evasion, and microenvironmental remodeling. Among them, *LBP* emerged as a top-ranked gene with strong mechanistic and clinical relevance, making it a compelling candidate for further functional validation in cellular models of KIRC. We next focused on evaluating the functional role of LBP, particularly in relation to tumor cell migration.

### Role of LBP in tumor cell migration

**Figure 7:**
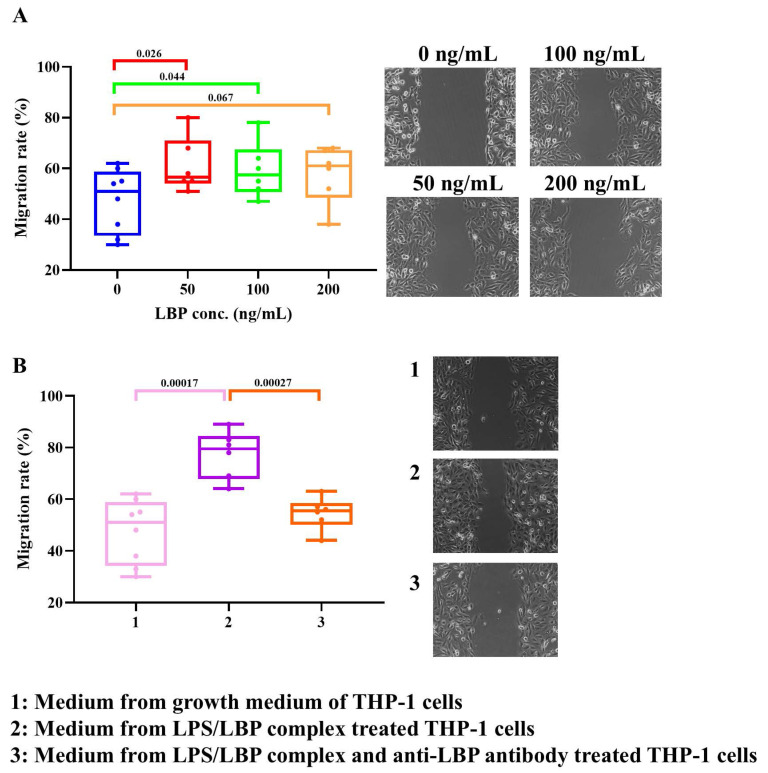
** LBP enhances 786-O cancer cell migration *in vitro*. (A)** Wound healing assay showing the effect of increasing concentrations of recombinant LBP (0-200 ng/mL) on cancer cell migration. Migration rate was significantly increased at 50 and 100 ng/mL. **(B)** Migration assay using conditioned medium from THP-1 cells. Medium from LPS/LBP-treated THP-1 cells promoted cancer cell migration, which was attenuated by the addition of anti-LBP antibody. Images on the right correspond to treatment groups: (1) control medium, (2) LPS/LBP-conditioned medium, (3) LPS/LBP with anti-LBP antibody.

Since high migratory activity is a hallmark of aggressive KIRC, we examined whether LBP regulates the migration of 786-O cells. Experimental results (Fig. 7A) demonstrated that LBP significantly promoted 786-O cell migration, with statistical significance observed at both low (50 ng/mL) and median (100 ng/mL) doses. Figure 1 shows that high immune infiltration, particularly macrophages, is associated with poor survival in KIRC patients. Notably, macrophages express multiple LBP protein receptors, suggesting a potential role for LBP in modulating tumor invasion. To investigate this, conditioned medium (CM) from human monocyte-like THP-1 cells was utilized to assess its impact on the migration of 786-O cells. As shown in Figure 7B, compared to standard growth medium, CM from LBP/LPS-treated THP-1 cells significantly enhanced 786-O cell migration. Moreover, when an anti-LBP antibody was added to the THP-1 culture containing the LBP/LPS complex, the pro-migratory effect of the CM was markedly reduced, indicating that LBP modulates tumor invasion through THP-1-secreted factors. ELISA results further revealed that LBP/LPS stimulation induced robust TNF-α and IL-6 secretion from THP-1 cells, while anti-LBP antibody suppressed this inflammatory response (Fig. S2), supporting LBP's regulatory role in the tumor microenvironment.

**Figure 8:**
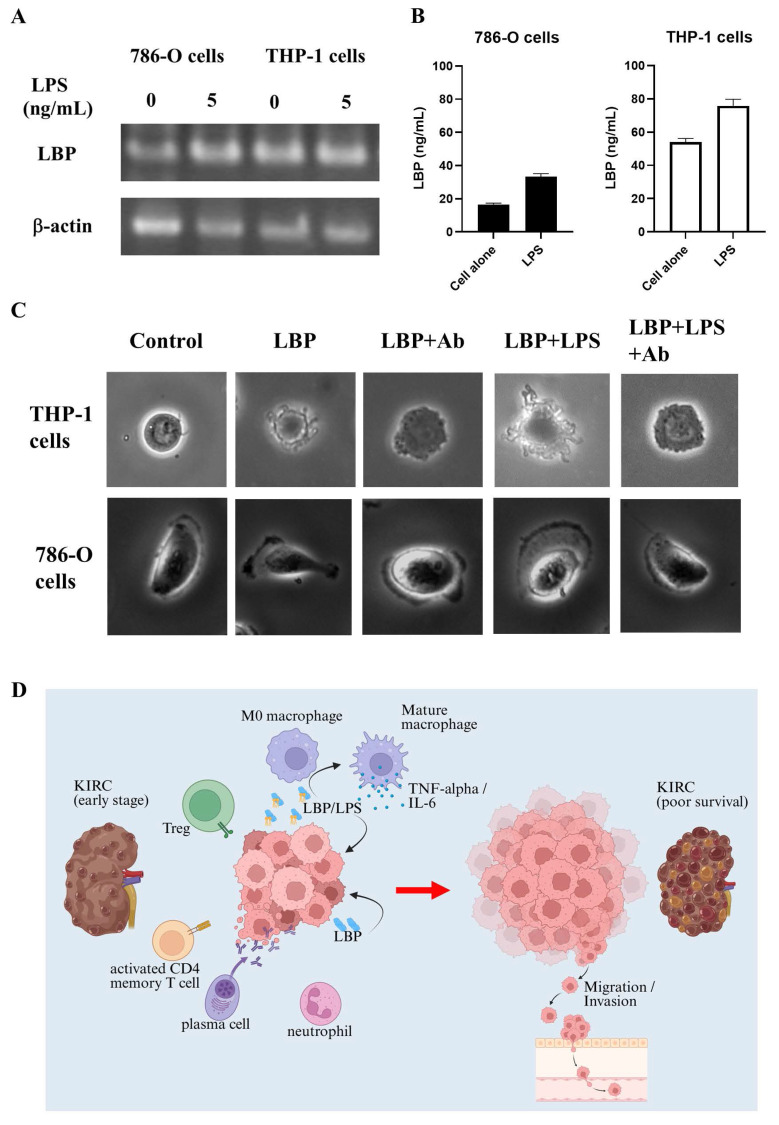
** LPS-induced LBP expression, its impact on cell morphology, and proposed model of LBP-mediated tumor progression in KIRC. (A)** RT-PCR analysis of LBP mRNA expression in 786-O and THP-1 cells following LPS stimulation (5 ng/mL). LBP expression was modestly increased in both cell types. β-actin served as an internal control. The lanes were run on the same gel but were non-adjacent and digitally rearranged. The grouping is indicated by white spaces.** (B)** ELISA quantification of LBP in cell culture supernatants confirmed LPS-induced upregulation in both 786-O and THP-1 cells. **(C)** Microscopy revealed morphological changes in 786-O and THP-1 cells following treatment with LBP or LBP/LPS, including altered cell shape and surface features. These changes were partially reversed by anti-LBP antibody, suggesting LBP-dependent modulation of cellular behavior. **(D)** Schematic model summarizing the proposed role of LBP in KIRC progression. LBP enhances macrophage activation and inflammatory cytokine secretion, promotes tumor cell migration and invasion, and contributes to the establishment of an aggressive, inflammation-driven tumor microenvironment associated with poor patient outcomes.

To delineate the cellular source of LBP, we quantified its transcripts and protein in 786-O and THP-1 cells by RT-PCR, ELISA, and Western blotting. 786-O cells showed only low LBP expression, whereas THP-1 cells displayed constitutive LBP expression that increased upon LPS stimulation. The dose-responsive rise in LBP protein detected by Western blot (β-actin loading control) is consistent with the RT-PCR/ELISA results (Fig. 8A, 8B, and S4).

Additionally, 3D imaging analysis (Fig. 8C) revealed that LBP treatment induced morphological changes in both THP-1 and 786-O cells. In the control group, THP-1 cells maintained a round shape with a smooth surface. For THP-1 cells, LBP exposure led to cell contraction and dendrite formation, which were partially inhibited by anti-LBP antibody, suggesting a role of LBP in modulating cellular morphology. Furthermore, when LBP was co-administered with LPS (LBP+LPS), the morphological changes became more pronounced, with irregularly extended cell dendrite appearing at the cell periphery. Notably, when anti-LBP antibody was added to the LBP+LPS-treated cells (LBP+LPS+Ab), the cells partially reverted to a more contracted state.

Similarly, in 786-O cells, LBP treatment promoted lamellipodia formation—a structure critical for motility—while anti-LBP antibody partially reversed this effect. These findings suggest that LBP influences tumor progression by modulating cell morphology and promoting inflammatory responses.

### Molecular mechanisms of LBP in KIRC

Figure 8D illustrates the proposed mechanism of LBP involvement in KIRC progression. In early-stage KIRC, immune cells such as M0 macrophages, Tregs, activated CD4 memory T cells, plasma cells, and neutrophils are present within the tumor microenvironment. Tumor cells secrete LBP, which binds to bacterial LPS, promoting the activation and maturation of M0 macrophages. These mature macrophages subsequently release cytokines, such as TNF-α and IL-6, which contribute to the remodeling of the tumor microenvironment. Notably, LBP can also function independently of LPS to directly enhance tumor cell progression. Through these mechanisms, LBP facilitates tumor growth, promotes immune suppression, and drives tumor cell migration and invasion—factors associated with poor survival in KIRC patients. These findings support the hypothesis that LBP is a key mediator of tumor aggressiveness and a potential therapeutic target in renal cancer.

## Discussion

This study delineates the immune and molecular characteristics associated with poor prognosis in KIRC. Through the integration of immune cell infiltration profiling, immune regulatory gene expression analysis, and functional validation of key mediators, we demonstrate that both immune suppression and chronic inflammation cooperatively shape the TME in aggressive disease.

Our findings provide additional insight into how specific immune cell populations influence KIRC prognosis. Among the 22 immune cell types analyzed, six—resting mast cells, Tregs, plasma cells, activated CD4 memory T cells, M0 macrophages, and neutrophils—were significantly associated with patient survival (Fig. 1). Notably, increased infiltration of Tregs and M0 macrophages in the poor-survival group suggests their potential utility as negative prognostic biomarkers, consistent with observations in other malignancies 26-29. Interestingly, not all findings conformed to classical immunological expectations. Activated CD4 memory T cells and plasma cells, typically linked to anti-tumor immunity, were paradoxically elevated in patients with poor prognosis. Similarly, HLA-related genes—normally associated with antigen presentation and immune activation—were also more highly expressed in this group (Table 1). These observations suggest a more complex immune regulatory environment in KIRC. Such features are increasingly recognized as hallmarks of immune exhaustion and functional reprogramming within chronically inflamed tumor microenvironments. We hypothesize that these seemingly contradictory patterns reflect an immunosuppressive shift within TME. Comparative analysis of tumor and adjacent normal tissues revealed a relatively higher gene index for the immunosuppressive cytokine TGF-β in tumors from poor-survival patients, while expression of interferon (IFN) family genes remained consistently low or undetectable (Table S1). This imbalance between immune activation and suppression implies a dampened anti-tumor immune response, potentially contributing to immune evasion and tumor progression. Furthermore, the elevated presence of activated CD4 memory T cells and plasma cells in poor-survival samples may reflect a functional reprogramming of these cells under chronic antigen exposure or the influence of immunosuppressive cytokines such as TGF-β and IL-10 30-33. Instead of mounting effective cytotoxic responses, these cells may adopt exhaust or regulatory phenotypes that support immune suppression or promote tumor progression. Plasma cells may also engage in immunoglobulin-mediated inflammatory signaling, indirectly contributing to a tumor-supportive environment 34, 35. In contrast, the abundance of Tregs, M0 macrophages, and TGF-β further establishes a microenvironment conducive to immune escape. Collectively, our findings depict an immunosuppressive yet inflammatory TME in poor-prognosis KIRC—a hallmark of immunologically “hot but functionally ineffective” tumors with impaired anti-tumor surveillance.

Further analysis identified LBP as a key regulator of tumor progression. While LBP has been traditionally recognized for its role in innate immunity, accumulating evidence indicates that it also participates in cancer-associated inflammation and immune modulation. Elevated LBP expression has been associated with poor prognosis in hepatocellular carcinoma, gastric cancer, and colorectal cancer 36-38, and genetic variants in the LBP gene have also been implicated in cancer susceptibility 37. In addition to its prognostic relevance, recent studies suggest that LBP may actively contribute to tumor progression by enhancing malignant phenotypes such as tumor cell proliferation, migration, and invasion, and shaping an inflammation-driven tumor microenvironment 17, 39, 40. These findings support a functional role for LBP beyond a passive biomarker, linking it more directly to tumor immunity and cancer-associated inflammatory signaling. Notably, intratumoral bacteria capable of producing lipopolysaccharide (LPS) may synergize with LBP to enhance tumor metastasis 41. Although LBP is classically recognized for its role in facilitating LPS-induced TLR4 signaling, accumulating evidence suggests that LBP may also participate in LPS-independent inflammatory processes. Previous studies have shown that LBP can interact with endogenous ligands, including phospholipids, and modulate immune signaling in the absence of bacterial components, thereby contributing to sterile inflammation. In addition, LBP has been reported to influence downstream inflammatory pathways beyond canonical TLR4 activation 42, 43.

In this context, our *in vitro* observations that recombinant LBP promotes tumor cell migration and induces morphological changes, while anti-LBP neutralization partially reverses these effects, are consistent with a model in which LBP may contribute to tumor progression through mechanisms that are not strictly dependent on bacterial LPS. Although previous studies lacked direct evidence of LBP production by renal cancer cells, our findings demonstrate that 786-O kidney cancer cells endogenously express and secrete LBP (Fig. 8). This tumor-derived LBP may act in concert with, or independently of, microbial signals to amplify inflammatory signaling within the KIRC tumor microenvironment, thereby accelerating disease progression. Functional assays revealed that LBP promotes cancer cell migration and modulates inflammatory responses. Conditioned media from LPS/LBP-stimulated THP-1 monocytes enhanced the migratory capacity of 786-O cells, suggesting that LBP may also exert indirect tumor-promoting effects via immune cell activation (Fig. 7). These effects were mitigated by an anti-LBP neutralizing antibody, which also induced notable morphological changes in both THP-1 and 786-O cells, reinforcing LBP's potential as a therapeutic target. Notably, such morphological alterations—particularly changes consistent with lamellipodia formation—have been widely associated with cytoskeletal remodeling and enhanced cell motility in response to inflammatory mediators, providing a morphological correlate to the increased migratory and metastatic potential inferred from our bioinformatics analyses. Moreover, analysis of the 20-gene interaction network (Table 1) using GeneMANIA implicated LBP in pathways related to extracellular matrix (ECM) remodeling, immune evasion, and inflammation-associated gene regulation (Fig. S3), which may explain its strong association with adverse clinical outcomes.

Chronic inflammation is a well-established hallmark of cancer development and progression. Cytokines such as IL-1, IL-6, and TNF-α are upregulated in response to inflammatory stimuli and stimulate hepatic synthesis of acute-phase proteins (APPs). These proteins play multifaceted roles in supporting tumor progression, including immune suppression, angiogenesis, and metastasis 44. For instance, fibrinogen (FG) facilitates pre-metastatic niche formation through ECM remodeling and pro-inflammatory signaling 45, while alpha-1 antitrypsin (AAT) promotes M2 macrophage polarization and inhibits cytotoxic T cell activity 46, 47. Elevated C-reactive protein (CRP) levels have been associated with poor prognosis in colorectal, breast, and renal cancers 47, and haptoglobin (Hp)—particularly its fucosylated form—is implicated in tumor progression across multiple malignancies, including breast, lung, and liver cancers 47-49. Beyond their utility as systemic inflammation markers, APPs reflect dynamic immune changes within the TME and may influence therapeutic outcomes. For example, increased Hp and ceruloplasmin (CP) levels have been associated with shorter progression-free survival in patients receiving ICIs 44. These observations highlight the interplay between chronic inflammation, immune modulation, and therapeutic response in cancer.

Although our primary goal was not to build a prediction tool, the resulting 19-gene signature demonstrated strong discriminatory power for poor survival in KIRC (Fig. 5) and was validated across external RNA-seq and microarray datasets (RECA-EU, E-MTAB-1980, GSE22541; Fig. 5). Notably, the panel retained moderate predictive capacity in other metabolically active or inflammation-prone cancers (PAAD, STAD, COAD), but not in LUAD, BRCA, or LIHC, underscoring its KIRC-specific context (Fig. 5). Although most genes within the 19-gene panel are not kidney-specific in terms of normal renal physiology, many are functionally involved in immune regulatory pathways that are highly relevant to renal cancer biology, including cytokine signaling, immune checkpoint regulation, and inflammasome/NF-κB-mediated inflammatory responses that characterize the KIRC tumor microenvironment (Table 2). The partial retention of prognostic performance in pancreatic, gastric, and colon cancers may reflect shared immune microenvironmental features with poor-prognosis KIRC. These tumor types are frequently associated with chronic inflammation, microbial exposure, and macrophage-dominated immune landscapes, all of which promote sustained innate immune activation. In such contexts, LBP may act as a mediator of cancer-associated inflammation by amplifying macrophage-driven cytokine signaling. In contrast, tumor types with less prominent inflammatory or innate immune components, such as LUAD, BRCA, and LIHC, showed limited applicability of the signature, highlighting its context-dependent biological relevance. This specificity strengthens the biological coherence of the gene panel and supports its relevance in KIRC-focused research.

Our study offers several contributions. First, it delineates the immune-suppressive architecture of poor-survival KIRC, supported by both cellular infiltration and molecular expression patterns. Second, it identifies LBP as a novel driver of tumor-promoting inflammation with both direct and paracrine effects. Third, it proposes a robust 19-gene immune signature derived from the same patient subset, reinforcing the mechanistic consistency of the observed phenomena. Importantly, while the 19-gene panel shows promise as a prognostic tool, its greater value lies in its biological insight—pinpointing candidate immune regulators that may underlie disease aggressiveness and therapeutic resistance.

## Conclusion

This integrated study reveals that immune suppression, inflammation, and tumor-immune crosstalk collectively contribute to poor prognosis in KIRC. LBP acts as a tumor-promoting effector linking bacterial signals and immune modulation, while the 19-gene panel offers a broader perspective on dysregulated immune processes in the TME. These findings open opportunities for personalized therapeutic strategies targeting the immune landscape of aggressive renal cancer.

## Supplementary Material

Supplementary figures and tables.

## Figures and Tables

**Table 1:** ** Top 20 immune regulation-associated genes prioritized by expression index in tumors of poor-survival KIRC patients.** Genes were ranked based on a composite index (expression × fold change), reflecting both high abundance and upregulation in tumor tissues relative to adjacent normal tissues from the same patients. The table includes gene symbols, corresponding protein names, average expression in tumors (n = 38), fold change compared to adjacent tissues, and detailed functional descriptions. Notable genes include *LBP* (lipopolysaccharide recognition), *COL1A1* (extracellular matrix remodeling), *FGA*/*FGB* (coagulation), and *C3* (complement activation), highlighting key immune-related processes associated with poor prognosis in KIRC. Functional annotations are curated from UniProt.

Gene name	Protein name	Gene expression	Fold change	Index	Function
LBP	Lipopolysaccharide-binding protein	102.86	82.72	8508.58	Acts as an affinity enhancer for CD14, facilitating its association with LPS. Promotes the release of cytokines in response to bacterial lipopolysaccharide
COL1A1	Collagen type I alpha 1 chain	281.4	16.56	4659.98	Type I collagen is a member of group I collagen (fibrillar forming collagen).
FGA	Fibrinogen alpha chain	119.28	38.54	4597.05	Cleaved by the protease thrombin to yield monomers which, together with fibrinogen beta (FGB) and fibrinogen gamma (FGG), polymerize to form an insoluble fibrin matrix. Fibrin has a major function in hemostasis as one of the primary components of blood clots.
FGB	Fibrinogen beta chain	243.86	17.23	4201.71	Cleaved by the protease thrombin to yield monomers which, together with fibrinogen alpha (FGA) and fibrinogen gamma (FGG), polymerize to form an insoluble fibrin matrix. Fibrin has a major function in hemostasis as one of the primary components of blood clots.
C3	Complement C3	284.99	12.39	3531.03	C3 plays a central role in the activation of the complement system.
FGG	Fibrinogen gamma chain	111.66	23.23	2593.86	Together with fibrinogen alpha (FGA) and fibrinogen beta (FGB), polymerizes to form an insoluble fibrin matrix. Has a major function in hemostasis as one of the primary components of blood clots.
CD74	HLA-DR antigens-associated invariant chain	1027.66	2.45	2517.77	Plays a critical role in MHC class II antigen processing by stabilizing peptide-free class II alpha/beta heterodimers in a complex soon after their synthesis and directing transport of the complex from the endoplasmic reticulum to the endosomal/lysosomal system where the antigen processing and binding of antigenic peptides to MHC class II takes place. Serves as cell surface receptor for the cytokine MIF.
HLA-B	HLA class I histocompatibility antigen, B alpha chain	840.9	2.89	2430.20	HLA-B belongs to the HLA class I heavy chain paralogues.
ACTB	Actin, cytoplasmic 1	1465.75	1.5	2198.63	Actin is a highly conserved protein that polymerizes to produce filaments that form cross-linked networks in the cytoplasm of cells
HLA-DRA	HLA class II histocompatibility antigen, DR alpha chain	857.02	2.5	2142.55	An alpha chain of antigen-presenting major histocompatibility complex class II (MHCII) molecule.
HLA-A	HLA class I histocompatibility antigen, A alpha chain	700.9	2.72	1906.45	HLA-A belongs to the HLA class I heavy chain paralogues.
B2M	Beta-2-microglobulin	878.18	2.02	1773.92	Component of the class I major histocompatibility complex (MHC). Involved in the presentation of peptide antigens to the immune system.
C1QB	Complement C1q subcomponent subunit B	180.01	7.75	1395.08	C1q associates with the proenzymes C1r and C1s to yield C1, the first component of the serum complement system. The collagen-like regions of C1q interact with the Ca2+-dependent C1r2C1s2 proenzyme complex, and efficient activation of C1 takes place on interaction of the globular heads of C1q with the Fc regions of IgG or IgM antibody present in immune complexes.
ENPP3	Ectonucleotide pyrophosphatase/phosphodiesterase family member 3	62.94	20.35	1280.83	Hydrolase that metabolizes extracellular nucleotides, including ATP, GTP, UTP and CTP
COL1A2	Collagen alpha-2(I) chain	177.99	7.03	1251.27	Type I collagen is a member of group I collagen (fibrillar forming collagen).
C1QA	Complement C1q subcomponent subunit A	195.03	6.36	1240.39	the same as C1QB
C1QC	Complement C1q subcomponent subunit C	174.56	6.88	1200.97	the same as C1QB
HLA-DRB1	HLA class II histocompatibility antigen, DRB1 beta chain	453.56	2.21	1002.37	A beta chain of antigen-presenting major histocompatibility complex class II (MHCII) molecule.
COL3A1	Collagen alpha-1(III) chain	206.08	4.47	921.18	Collagen type III occurs in most soft connective tissues along with type I collagen. Involved in regulation of cortical development.
HLA-C	HLA class I histocompatibility antigen, C alpha chain	484.89	1.82	882.50	HLA-C belongs to the HLA class I heavy chain paralogues.

**Table 2:** ** Functional annotation of the 19-gene panel associated with poor prognosis in KIRC.** For each gene in the final 19-gene panel, both the canonical biological function and its refined role in the context of kidney renal clear cell carcinoma (KIRC) and immune regulation are summarized. Annotations integrate information from public databases (e.g., UniProt, GO) and literature review, with emphasis on immune modulation, inflammation, and tumor progression. Several genes (e.g., *IL4*, *RNF135*, *NLRC3*, *NOD2*, *HHLA2*) are involved in key immune pathways such as cytokine signaling, immune checkpoint regulation, and inflammasome activation, highlighting their potential roles in shaping the tumor microenvironment and influencing clinical outcomes in KIRC.

Gene name	Original function description	Refined function (KIRC/Immune context)
IL4	Cytokine involved in the differentiation of naive helper T cells (Th0 cells) to Th2 cells.	Promotes Th2/Treg polarization, suppressing antitumor immunity.
RNF135	E3 ubiquitin ligase involved in innate immune response and potentially cell proliferation.	Enhances tumor survival through PI3K-AKT signaling and involved in β-catenin activation.
NLRC3	Negative regulator of the PI3K-AKT-mTOR signaling pathway.	Inhibits mTOR signaling; its downregulation facilitates tumor progression.
NOD2	Recognizes bacterial molecules; triggers immune responses via NF-kB activation.	Triggers NF-kB signaling and inflammasome activation in macrophages.
HHLA2	Modulates T cell function; part of B7 family of immune checkpoint molecules.	Immune checkpoint molecule; downregulation linked to immune escape.
BAX	Promotes apoptosis; regulates programmed cell death.	Regulates apoptosis; associated with increased T cell death in tumors.
EIF2B1	Subunit of a translation initiation factor; involved in protein synthesis and stress response.	Facilitates protein synthesis and stress adaptation in immune cells.
KIR2DL4	Killer cell immunoglobulin-like receptor; involved in NK and T cell activation.	NK and T cell receptor; downregulation weakens cytotoxic responses.
SRC	Non-receptor tyrosine kinase involved in the regulation of cell growth and differentiation.	Promotes tumor proliferation and metastasis via RAS-RAF-ERK pathway activation.
IFNL1	Antiviral cytokine; contributes to the immune defense against viruses.	Antiviral cytokine; loss reduces immune-mediated tumor clearance.
PDE4B	Enzyme that hydrolyzes cAMP; regulates cellular responses to hormones and neurotransmitters.	Modulates cytokine release and immune response via cAMP degradation.
GPS2	Inhibits inflammatory gene expression; involved in transcriptional regulation.	Suppresses inflammatory gene transcription; linked to macrophage regulation.
HAVCR2	Encodes TIM-3; a checkpoint receptor involved in immune regulation.	Encodes TIM-3 checkpoint; immune exhaustion marker on T cells.
OSCAR	Activating receptor on myeloid cells; promotes immune responses.	Amplifies myeloid cell activation and inflammation in TME.
GRN	Secreted protein that is involved in cell growth, survival, and repair processes.	Supports tumor growth and tissue remodeling; secreted growth factor.
POLR3G	Subunit of RNA polymerase III; contributes to the transcription of small RNAs including tRNAs and 5S rRNA.	Drives non-coding RNA expression linked to tumor invasiveness.
IL27RA	Receptor subunit involved in IL-27 mediated signaling; modulates immune responses.	Mediates IL-27 signaling that enhances Treg cell activity.
BTNL3	Member of the butyrophilin family; may regulate immune cell proliferation.	Modulates T cell proliferation, contributing to immune suppression.
FCGR1A	High-affinity IgG receptor; activates phagocytosis and cytokine release.	Activates macrophage phagocytosis and proinflammatory cytokine release.

## Data Availability

The datasets used and/or analyzed during the current study are available from the corresponding author upon reasonable request.

## Abbreviations

KIRC: Kidney renal clear cell carcinoma
LBP: Lipopolysaccharide-binding protein
TCGA: The Cancer Genome Atlas
GO: Gene Ontology
TME: Tumor microenvironment
LPS: Lipopolysaccharides
ICIs: Immune checkpoint inhibitors
ECM: Extracellular matrix
APPs: Acute-phase proteins
GMM: Gaussian Mixture Model
