# Supplementary material for: A prognostic 19-gene signature and LBP-mediated immune dysregulation define the tumor microenvironment in poor-prognosis KIRC
Source: Int J Med Sci. 2026 Jan 30;23(3):824–42. doi: 10.7150/ijms.125505 (PMC12964576; doi:10.7150/ijms.125505)
Supplement: Supplementary file 1 — Supplementary figures and tables. [file ijmsv23p0824s1.pdf]

Supplementary Results

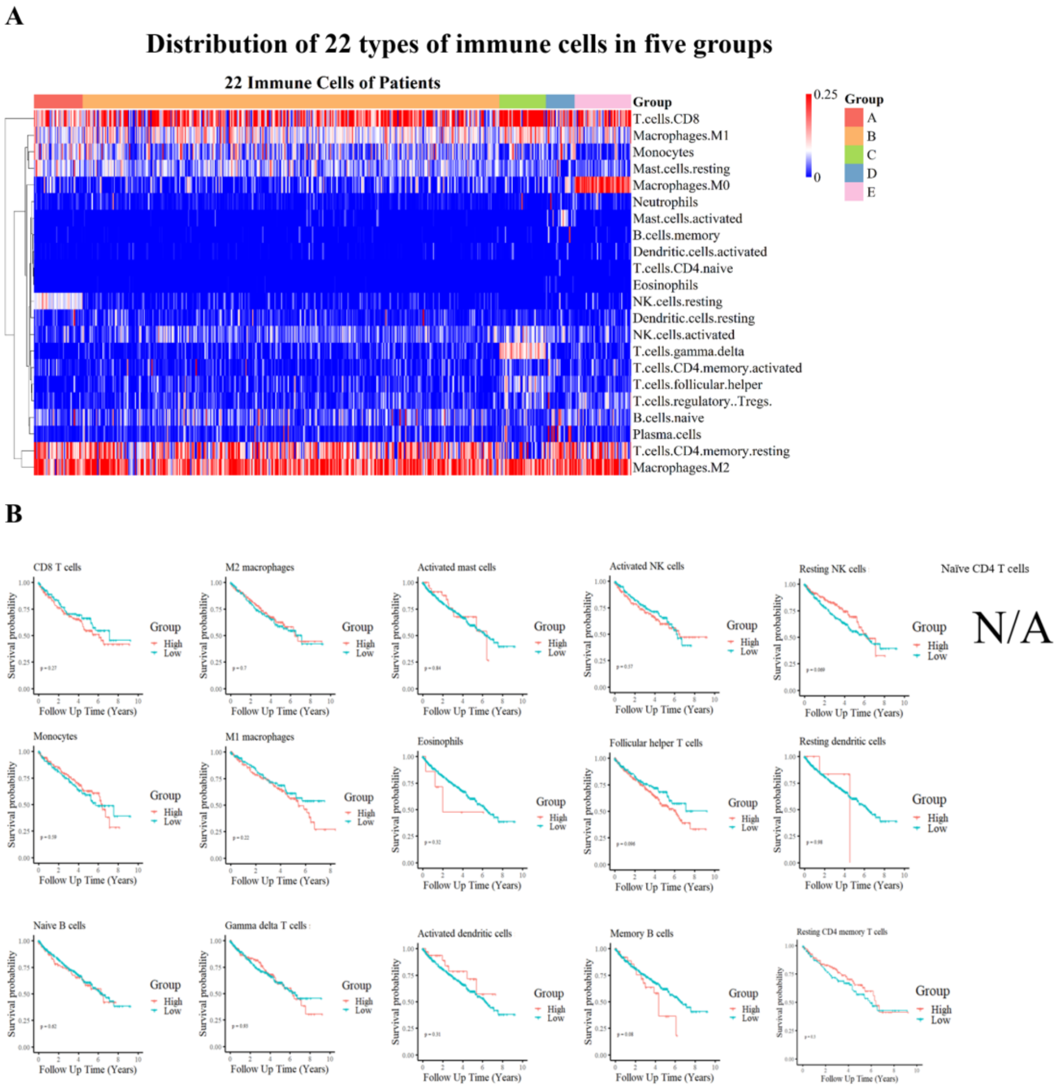

Figure S1. Immune cell distribution and prognostic analysis of remaining immune cell types. (A) Heatmap showing the abundance of 22 immune cell types across five immune subgroups (Groups A–E). **(B)** Kaplan-Meier analysis of 16 immune cell types not shown in Figure 1C. Most were not significantly associated with survival. Naïve CD4 T cells were excluded from analysis due to insufficient data (marked as N/A).

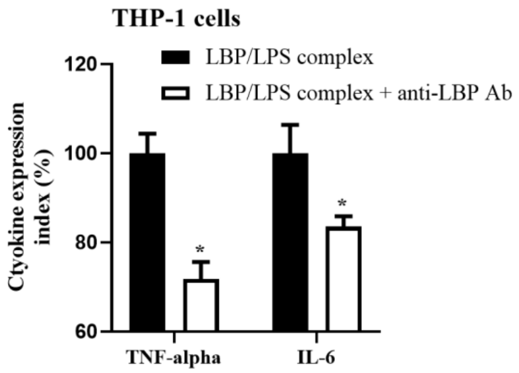

Figure S2. Anti-LBP antibody reduces cytokine production in THP-1 cells. Bar graph showing expression levels of TNF-α and IL-6 in THP-1 cells treated with LBP/LPS complex, with or without anti-LBP antibody. Neutralization of LBP significantly reduced cytokine levels (*$p < 0.05$), supporting its role in macrophage activation.

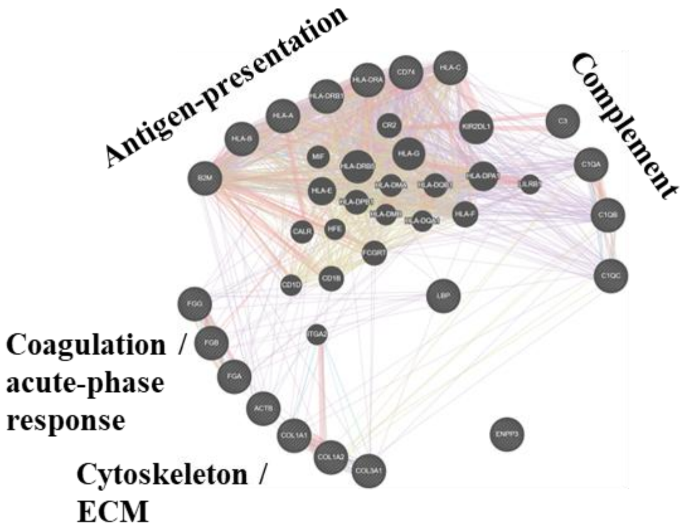

Figure S3. Functional gene interaction network of 20 candidate genes generated by GeneMANIA. GeneMANIA was used to visualize functional associations among the top 20 immune-related genes (Table 1) identified in poor-survival KIRC tumors. The resulting network revealed that LBP was centrally positioned and connected to multiple gene clusters, suggesting its critical role in bridging immune responses and extracellular matrix (ECM) remodeling. Specifically, LBP-associated gene clusters were found to be primarily involved in antigen presentation, complement activation, ECM remodeling, coagulation, and acute-phase response. This network highlights LBP as a central regulatory molecule linking inflammatory responses, immune modulation, ECM remodeling, and coagulation pathways. Its interactions suggest that LBP might contribute to both immune evasion and tumor progression by modulating the tumor microenvironment.

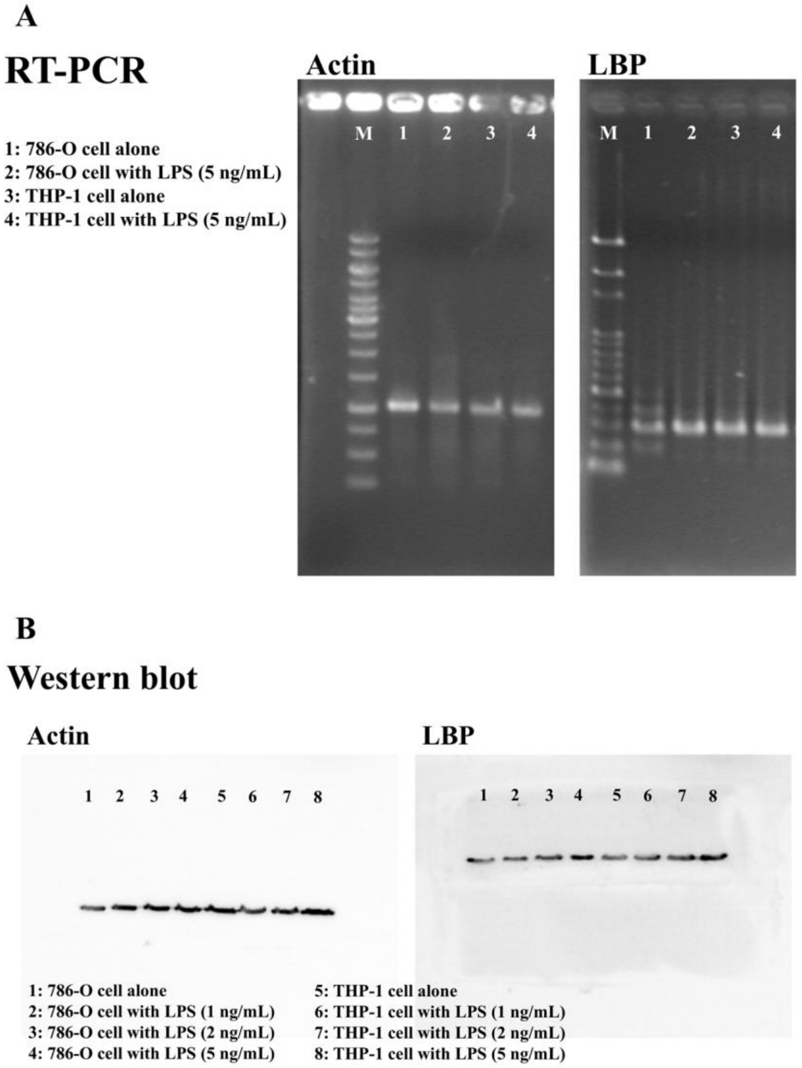

Figure S4. RT-PCR and Western blot validation of LBP expression in 786-O and THP-1 cells following LPS stimulation. (A) RT-PCR was evaluated for LBP expression in human monocytic THP-1 cells and human renal carcinoma 786-O cells, with β-actin as the internal control. **(B)** Western blot analysis was performed to detect LBP expression in 786-O cells (lanes 1–4) and THP-1 cells (lanes 5–8) under different concentrations of LPS stimulation. β-actin was used as a loading control. Lane assignments: (1) 786-O cell alone, (2) 786-O cell with LPS (1 ng/mL), (3) 786-O cell

with LPS (2 ng/mL), (4) 786-O cell with LPS (5 ng/mL), (5) THP-1 cell alone, (6) THP-1 cell with LPS (1 ng/mL), (7) THP-1 cell with LPS (2 ng/mL), (8) THP-1 cell with LPS (5 ng/mL). LBP expression increased in a dose-dependent manner upon LPS stimulation in both 786-O and THP-1 cells, confirming the transcriptional results obtained by RT-PCR.

Table S1. Poor survival of KIRC gene index

Gene name	Index
LBP	8508.686056
COL1A1	4661.130434
FGA	4596.918517
FGB	4202.196315
C3	3531.555545
FGG	2593.340145
CD74	2516.250766
HLA.B	2431.246916
ACTB	2205.528974
HLA.DRA	2140.500782
HLA.A	1903.41451
B2M	1770.745408
C1QB	1395.809295
ENPP3	1281.023656
COL1A2	1251.978643
C1QA	1240.09554
C1QC	1200.198144
HLA.DRB1	1002.320057
COL3A1	921.6284234
HLA.C	884.1039632
HMOX1	870.3812464
ACTG1	764.3246786
RPS19	734.4102
TYROBP	718.3470645
TREM2	644.2716581
CLU	595.1396991
C1S	557.9989512
APOE	536.153289
HLA.E	530.3836623
FCGR3A	528.7622274
C1R	481.8539622
UBC	480.291512
FCER1G	458.3122811

SERPING1	446.9283798
CTSB	425.4565086
IL20RB	422.929023
CD24	414.482217
CAV1	402.2979608
HLA.DPB1	402.2904475
HSP90B1	390.1222581
BST2	369.4512311
HSP90AB1	348.2216454
LGALS3	319.9742852
S100A9	292.4625222
CD14	275.440079
CD177	274.1817458
CCL5	255.2484413
RPS3	248.4403421
RPS27A	201.3066478
HLA.DRB5	185.4600169
HLA.DPA1	185.3382099
TGFB1	182.4396041
A2M	180.866075
IGLL5	177.3413314
CD99	173.8470979
PROS1	172.2787705
HLA.G	164.2429055
VCAM1	163.5423608
CTSS	161.1886547
JUN	158.0414334
GRN	157.536391
CTSL	156.6133807
IFITM1	153.4812751
CYBA	149.5401947
FKBP1A	149.3859436
SPON2	145.2307174
SOCS3	144.0495422
SHC1	138.537654
PSMB8	136.9613185

ARPC1B	136.9417666
ANXA1	135.5329739
CTSK	130.2759136
UBA52	130.1967027
S100A8	127.7944021
CLEC2B	125.0000725
UBB	124.4482497
PSMB4	123.0617499
RBCK1	122.9625712
ARPC3	121.6904182
RAC1	121.6812529
ITGB2	119.5262235
FOS	118.1730892
BIRC3	117.5006611
SLAMF8	115.0570775
ICAM1	114.4727993
HLA.DQA2	107.402148
RGCC	106.0053752
CD81	105.8387824
HLA.F	104.5932831
IFI16	104.5272126
ITGB1	103.3181972
NOP53	102.8322177
HLA.DQA1	100.0186864
EZR	99.26807045
LY96	97.01459434
ARPC1A	96.79222972
VAMP8	94.80891985
MIF	92.16019925
PSMB3	91.67724326
CNPY3	90.22714428
CMTM3	87.50058076
PSMA7	86.75006762
PHPT1	85.89858285
CD300A	84.28733441
PSME1	84.01332744

NFKBIA	83.63144789
ACTR2	82.3055184
FLOT1	81.62673471
C2	80.7729155
IFNGR2	80.54236218
TNIP1	78.23407275
VSIG4	76.96515977
RAC2	75.9843726
DNASE2	75.13956822
GRAMD4	75.05487024
CD4	74.6435578
CD59	74.09374288
PYCARD	73.96271062
HSPD1	73.20774967
IFNGR1	72.31307095
PSMB7	71.58723628
GBP1	70.34904289
FPR3	68.78816419
C5AR1	68.42132735
LGMN	67.78889652
CD40	67.05590456
PSMB9	65.941066
PSMB1	64.19507274
HSP90AA1	63.25638672
BRK1	62.7358943
THY1	62.05473187
PARK7	61.26769227
CDC37	61.02356231
CFH	59.95518451
HHLA2	59.21608779
IRF7	59.13254329
BAX	59.09748618
S100A1	58.60474578
HLA.DQB2	57.7242713
PKN1	57.36217044
PSMC3	56.19471827

ADA	55.25697797
XRCC6	55.06301831
STAT1	54.61222748
CFI	54.59381174
FCGRT	54.14175735
SAMHD1	54.05144627
PSME2	53.87185931
PSMD8	53.86397303
TNFSF13B	52.94745309
LGALS9	52.46527216
PRNP	52.14856868
TNFRSF1B	52.00877066
CFD	51.93963514
PSMB6	50.47062093
CDC42	49.99833947
CD276	49.71068208
PTPN1	49.67821635
C3AR1	49.56353554
NONO	49.08463122
MMP12	46.85654568
PSMD4	46.23797652
FCGR2A	46.22229122
UBE2D2	45.92347917
TNFAIP3	45.88518816
HAVCR2	45.76564989
PSMB5	45.41035351
THEMIS2	45.03928839
TNFRSF14	43.56204527
BTN3A2	43.39538406
ARPC2	42.97749763
NR1H4	42.87778661
MAPKAPK2	42.08247816
HCST	41.68090965
JAK3	41.58077222
SASH3	40.76445204
STAT6	40.44988881

CD3D	40.40481333
IRAK1	40.32738591
C4B	39.99984382
PSMB10	39.32826618
ARRB2	39.17880837
LAIR1	39.04882771
F2	38.38048571
CXCL13	38.13326286
STING1	37.75808509
RTN4	37.68292311
NPDC1	37.68243471
HCK	37.39954018
CD79A	37.33154775
CARD11	37.09362019
IL4R	37.03075037
STX4	36.45534962
CD8A	35.66356981
PQBP1	35.60160949
CALM1	35.58039772
ADAM8	35.45809202
IL6ST	35.20522748
WAS	34.26407191
CD3E	34.24213301
XRCC5	33.9324783
C1RL	33.92539645
GRB2	33.74344025
ARPC5	33.71325647
PSMD2	32.79252299
KARS1	32.74892098
PSMC4	32.72956634
SUMO1	32.04136891
PSMA1	32.04085337
PIGR	32.03943127
PJA2	31.56471626
ARPC4	31.01222906
CD200	30.95925375

STOML2	30.89885573
DDX3X	30.82818446
LAMP1	30.76767852
JAK1	30.61593319
MNDA	30.32731329
NR1H2	30.14209211
LCP2	30.10784325
PSMC2	29.59476971
BAG6	29.56274523
ECM1	29.52561426
MYO1C	29.4899653
CD36	29.3406239
C4A	28.9134356
RUNX1	28.82735784
CUEDC2	28.76983265
BCL6	28.76982445
PIK3AP1	28.68306278
PSMF1	28.62138586
PSMD13	28.38628286
IL6	28.36166203
PLSCR1	28.17332153
WASF2	27.77517395
IDO1	27.67896932
FYB1	27.59143764
BTN3A1	26.80509874
VSIR	26.7844489
LSM14A	26.59924306
SFPQ	26.50372443
CD86	26.49619422
RARA	26.32759437
WIPF1	26.03702383
LILRB4	25.88431816
ACTR3	25.62505017
FPR1	25.5187028
PTPRC	25.51018717
ADAR	25.48539463

APOB	25.35115736
HLA.DMB	25.1642676
NECTIN2	24.72729836
HK1	24.6211167
CFB	24.60217363
HRAS	24.58998454
HMGB2	24.50452421
PILRA	24.4762326
PVR	24.37612044
TLR2	24.05928473
CUL1	24.05285023
NRAS	24.04166036
RIPK2	23.98210412
CBFB	23.6591147
CD34	23.41507177
LIMK1	23.32942762
UBE2D3	23.15724007
PSMB2	23.08191192
IRF3	23.03359037
HMGB1	23.00653861
PSMA3	22.61806041
FGR	22.47450936
LPXN	22.41298119
NFKBIZ	22.34700291
PSMD3	22.32836114
IL27RA	22.06855746
HLX	22.06082104
NR1H3	21.79395844
CSK	21.60075185
PARP14	21.1768703
TGFB3	21.05084975
NCKAP1L	20.89043182
NDFIP1	20.79975847
PTPN6	20.79799816
TLR3	20.77595102
C1QBP	20.66107887

SERPINB9	20.62197621
MAPK3	20.31015453
TNIP2	20.30674734
PSMD7	20.25423164
HSPA1A	20.22926607
ERAP1	20.21710382
PSMC5	20.01171918
PAK2	19.97604723
CRK	19.81091926
ITGAL	19.70917603
RFTN1	19.32998254
ITGAM	19.18549616
TNFRSF21	19.05400904
TICAM1	19.03516208
RAPGEF1	18.98031203
SEM1	18.88109379
SPG21	18.8451885
RELA	18.83719238
HSPA1B	18.81311252
PSMD10	18.25276084
DDX21	18.23969165
IL18	18.0330685
WDFY1	17.90317455
CLEC4E	17.82435355
SLAMF7	17.77226123
RELB	17.53181987
PPP3R1	17.22878131
DUSP3	17.14712944
MYD88	17.09761727
CRKL	17.06530851
UNC93B1	17.02948194
PAG1	16.85309533
EIF2B1	16.79434291
DDX1	16.79284806
RNF135	16.76106225
CD48	16.60338431

YTHDF3	16.29136441
WASL	16.2505432
PSMA4	16.19538399
PRKD2	15.94495893
IL1R1	15.82273423
MAPK1	15.7990429
PTAFR	15.73338422
C6	15.65347183
ABL1	15.57780114
CD46	15.45244618
RIOK3	15.01291853
CD84	15.00950003
NPLOC4	15.00793883
NR1D1	14.99820436
NFKBIL1	14.9795884
NMI	14.92640174
OSCAR	14.90810516
SKP1	14.77089006
BIRC2	14.70252325
PTPN11	14.64547904
LILRB2	14.48633792
NCK1	14.47080286
TFRC	14.36613264
PUM2	14.31265073
RAP1A	14.27762953
FCGR1A	14.13954097
PELI1	13.97049533
CD47	13.96875693
LYN	13.75167718
IRF1	13.62404231
YTHDF2	13.59523289
ZBTB7B	13.55795197
IL7R	13.49625744
SELENOS	13.47701997
PHB	13.4456259
SRC	13.44220541

MEF2C	13.3064714
DHX9	13.29103719
UBE2N	13.22088277
PRKACA	13.15338274
NLRC5	13.01844339
LAT2	12.89545418
KLRB1	12.88740056
HMCES	12.68646459
PLCG1	12.67896711
ABI1	12.64316802
PSMD11	12.63690665
PSMD12	12.62921137
INPP5D	12.62501597
UBE2K	12.60726533
CD274	12.59056685
PSMD1	12.57364471
VAMP7	12.43989472
ELF1	12.39937773
RIPK1	12.28923969
SPPL2B	11.84778701
IFNAR2	11.75668748
FAM3A	11.74843184
TREM1	11.68060423
GBP5	11.56429175
PRKCD	11.32240846
POLR3C	11.16485435
UBQLN1	11.12997094
BCAR1	11.04628089
SPPL3	10.94210654
PIK3R1	10.90920665
CYFIP1	10.90568911
BTN2A1	10.89970037
SEMA7A	10.87959851
BTN3A3	10.87776215
PARP9	10.81760764
YES1	10.76552227

PSMA5	10.74752489
CD1D	10.65727836
FYN	10.6351879
DUSP10	10.53901971
TRAFD1	10.53493441
COCH	10.38031771
CD55	10.37887258
HEXIM1	10.30687494
CLCF1	10.25618294
STAT5B	10.23241302
SNX4	10.20786695
NFAM1	10.20735998
MASP1	10.20525725
TRAF2	10.13168757
VAV1	9.943271413
CLEC4A	9.871978269
SEC14L1	9.871102767
SELL	9.830521231
NCKIPSD	9.797949929
ABCE1	9.794181552
MUC1	9.769189465
CASP8	9.640908286
PSME3	9.594312985
IKBKE	9.504896473
PLD2	9.472377988
P2RX7	9.449050317
TBK1	9.443400574
AGER	9.422348364
STXBP2	9.268156345
ELMO1	9.222783783
CTSH	9.195411121
DDX58	9.18754587
PSMD14	9.137559902
MALT1	9.109394284
ZP3	9.099534681
CD300C	9.085671859

UBE2D1	9.068139459
SUPT6H	9.053205535
ZC3H12A	9.013364828
PPP3CA	8.999543845
PUM1	8.91864647
RPS6KA3	8.883782562
TTLL12	8.63174159
FZD5	8.53838097
ITGA4	8.495960154
DAB2IP	8.427230034
NFKB1	8.404630654
MAVS	8.357581184
SOCS1	8.279932133
MAD2L2	8.21346092
CMKLR1	8.202280159
ERBIN	8.165684573
ZCCHC3	8.132103684
TANK	8.090736223
HFE	8.06395579
BCL10	8.036059692
MUL1	7.986352825
TAB2	7.981918101
EIF2B5	7.854694144
IL18R1	7.742710143
CYRIB	7.725715849
SLC11A1	7.714775775
CRP	7.651813063
FBXW11	7.634354729
IFIH1	7.622619137
SMAD7	7.600964218
MYO1G	7.575268531
SAMSN1	7.569415278
TAB1	7.552493241
MAPK14	7.527355713
RAB7B	7.466209434
ITCH	7.436634508

CNOT7	7.393822506
SH2B2	7.303452107
PTK2	7.288892557
LAG3	7.264125421
TSPAN6	7.222889656
LOXL3	7.201690368
BCL2	7.149918283
MAPKAPK3	7.14583348
PAK1	7.131163009
CPN2	7.082681627
EIF2B4	7.071464163
TRIM5	7.006889464
RBM14	7.003674115
ABR	6.994847827
NFATC2	6.928552217
SLAMF6	6.921101079
F2RL1	6.909029017
EP300	6.903446897
COLEC12	6.883334456
SOS1	6.867083593
DDX60	6.82306208
TRIM27	6.75272978
CD300LF	6.727988427
UFD1	6.723968478
MAP2K7	6.717689621
MUC3A	6.715377791
OTULIN	6.597208194
PLEKHA1	6.573972313
FES	6.538389779
POLR3D	6.528375178
PSMD5	6.515228791
SPPL2A	6.503163893
PTPRS	6.501702398
MICB	6.43415997
WIPF2	6.419039991
RAF1	6.408694347

TLR4	6.381694188
PTPN2	6.368108994
PSMC6	6.365639406
PPP3CB	6.362494721
PIK3CD	6.33758807
CYLD	6.315959439
FCN1	6.229910579
EXOSC6	6.193367767
LGR4	6.088002708
AP1G1	6.080191136
LCK	6.077503233
MAPK9	6.001722208
FCN3	5.990304532
OTUD4	5.903811938
PRKDC	5.859847653
SIGLEC9	5.850881523
PARP3	5.798845754
NCKAP1	5.796917856
APPL2	5.776121056
SOCS5	5.748068828
ZBTB1	5.70826898
CLEC2D	5.668491925
VAV2	5.656840068
BTNL9	5.655065772
EIF2B2	5.653313452
DHX58	5.590305796
TLR7	5.57751002
GAB2	5.563813837
MAP3K7	5.554462081
XIAP	5.535224483
SIRT1	5.519166657
BTN2A2	5.518505282
IL13RA2	5.501505279
FADD	5.472656883
KRAS	5.46881227
NFKBID	5.454532481

FGL2	5.450138824
CREBBP	5.374625319
TRIM15	5.309196108
PSEN1	5.261704204
BTK	5.257446577
STK11	5.23833181
PIK3CB	5.187763399
DUSP22	5.157087007
PPP2R3C	5.152722464
IL33	5.148475054
PRKCH	5.088733157
SLC39A10	5.044488367
PSME4	5.032602754
SMAD3	5.027811307
MLH1	5.025518773
EIF2AK4	5.004069763
CACNB3	4.99744588
ELMO2	4.938081158
C8G	4.88354333
PRKACB	4.861675945
PSMA2	4.77300954
IRAK4	4.762598948
C12orf4	4.724302898
CD247	4.697866071
ATG5	4.680138471
ANKRD17	4.667478607
RSAD2	4.622854593
PSPC1	4.560822916
PTPRJ	4.53569471
IL2RA	4.492529613
ARG2	4.47859758
GPATCH3	4.457699394
MSH2	4.450413626
CD79B	4.383673805
PDE4B	4.367336803
MAP2K4	4.353709971

APPL1	4.345177226
POLR3F	4.340663957
FBXO38	4.334906076
STX7	4.324792846
WNK1	4.298530658
ADGRE2	4.281641719
DOCK1	4.238365046
METTL3	4.229418258
HMGB3	4.12961478
SKAP1	4.112543967
FCGR2B	4.105935564
EIF2B3	4.083460554
SHLD2	4.057541847
MUC20	4.042471821
LAIR2	4.040324154
ICAM2	4.017190412
RAB29	4.010482661
JAML	4.001954919
PLA2G6	3.999088332
IL1B	3.99329469
UNC13D	3.95146987
PPARG	3.874162062
MED1	3.847860685
JAK2	3.839025893
DHX36	3.810500191
KMT5B	3.793094016
CCL19	3.792455542
CD8B	3.788366341
STAP1	3.766686447
CCR7	3.703734233
KIT	3.677427029
AZGP1	3.657546693
TRAF3	3.597075892
LRRC14	3.560560334
EXOSC3	3.543636564
THOC1	3.485062363

VTN	3.444645759
FOXP1	3.410605237
IKBKB	3.408121294
PIK3R4	3.406745339
SIGLEC7	3.38085955
KMT5C	3.374341517
CD96	3.374009723
SYK	3.37217983
NOD1	3.364569438
RIF1	3.339963725
SIN3A	3.330937826
PSMD6	3.314880475
SPNS2	3.311940919
IRAK2	3.292330658
BRAF	3.282999987
PSMA6	3.280220136
ELF2	3.278823338
FOXF1	3.274933939
NFATC1	3.163640858
ERMAP	3.154761493
LTF	3.132757834
CCR2	3.112491226
TLR1	3.110107846
CACTIN	3.103087794
RC3H2	3.082111928
RC3H1	3.042523125
TNFSF13	3.027890967
WIPF3	3.013317693
CD300E	2.986623612
PIAS1	2.92452913
IRAK3	2.916070051
ALPK1	2.90642225
NR4A3	2.8885929
PIK3R6	2.856544822
MAP3K1	2.849173988
PIK3CA	2.842947721

NLRX1	2.802827928
STXBP1	2.770693917
GPS2	2.762603898
AIM2	2.750403448
SH2D1A	2.741542241
NLRP3	2.699372657
SCIMP	2.66885548
USP15	2.665189429
ADORA2B	2.657041522
PIANP	2.647911713
SLA2	2.624081385
PDE4D	2.6173854
C4BPA	2.563230289
TNFSF4	2.556565412
CGAS	2.536128525
CD209	2.524363565
TIFA	2.506788812
RASGRP1	2.49632171
SMPDL3B	2.480759953
TRAF6	2.476886152
MYO10	2.474446641
NSD2	2.46104305
TLR8	2.448260629
RAB11FIP2	2.44729267
TRIM6	2.308336375
MUC13	2.280647254
KLHL6	2.256255739
PSMD9	2.240593515
TRPM4	2.199822667
RNASEL	2.170899302
SHLD1	2.165137982
VAV3	2.161304001
PDCD1	2.153949191
RNF31	2.109976079
ZAP70	2.10437174
PSMC1	2.079844861

UBE2V1	2.073640207
GPRC5B	2.067936787
TP53BP1	2.063647547
PAWR	2.038645599
CD3G	2.002051491
KCNN4	1.984899864
CHUK	1.982678324
TAB3	1.962778551
INAVA	1.948547177
MUC17	1.883715053
CADM1	1.880407773
CD5L	1.879370882
PDPK1	1.87785234
BCR	1.871973686
IL12RB1	1.859848762
BTRC	1.859191321
TRIL	1.838055515
NFATC3	1.829983111
RNF125	1.799673813
MAPK8	1.784743845
PLCL2	1.776194665
CXADR	1.774070219
BAIAP2	1.733320784
USP18	1.709517661
PAXIP1	1.697326046
CLEC10A	1.697199327
MFHAS1	1.671361866
PIK3C3	1.660461321
CD28	1.654735726
PTPN22	1.517015785
PRAM1	1.500854887
CYFIP2	1.495653499
PILRB	1.477125443
FOXP3	1.473566075
ITGB7	1.453417711
NLRC3	1.449434948

IL15	1.438451771
EPG5	1.424542566
TKFC	1.416349137
IKBKG	1.415245554
PLCG2	1.398103589
TIRAP	1.343659121
LIME1	1.309541504
NLRC4	1.300314034
WNT5A	1.27212596
CD38	1.240051942
CD40LG	1.179157427
FER	1.155453413
SHLD3	1.155278676
ADCY7	1.105802571
CD300LB	1.101341671
COLEC11	1.063932918
PLA2G1B	1.062286365
ANGPT1	1.055101599
KLRF1	1.026418799
RIPK3	1.02525332
PRKCB	1.017532041
SARM1	1
PGLYRP1	1
MASP2	1
CD22	1
MATR3	1
C8B	1
TNIP3	1
COL17A1	1
TBX21	1
TXK	1
ICAM3	1
PAK3	1
CPB2	1
CFHR2	1
ESR1	1

PGC	1
NCR2	1
MADCAM1	1
XBP1	1
GRAP2	1
CTSG	1
RPS6KA5	1
CACNA1F	1
ACOD1	1
FGL1	1
FCER2	1
LILRA1	1
CLC	1
ICAM4	1
ICAM5	1
CD33	1
PIK3R2	1
IL2	1
CRTAM	1
HPX	1
IL23A	1
IFNG	1
ULBP1	1
TREML2	1
GPLD1	1
ITK	1
IL12B	1
BTNL8	1
POLR3G	1
IL4	1
C9	1
IL18RAP	1
ZP4	1
CD160	1
MUC5B	1
APOA1	1

CNR1	1
MYB	1
ARG1	1
SLC46A2	1
CPN1	1
GPR31	1
CD80	1
LAX1	1
SFTPA1	1
C4BPB	1
ZBP1	1
BTN1A1	1
EREG	1
MYH2	1
KIR2DL1	1
BPIFB1	1
FFAR2	1
CFP	1
PLA2G5	1
IGLL1	1
SPINK5	1
CFHR4	1
CFHR5	1
KLRD1	1
KLRC1	1
C5AR2	1
TESPA1	1
TEC	1
BLK	1
IL10	1
IRF4	1
IL21	1
COL2A1	1
ADCYAP1	1
GPR32	1
XCL1	1

REG3G	1
GPR17	1
MUC4	1
IFNK	1
DRD2	1
MS4A2	1
CD226	1
PSMA8	1
MS4A1	1
C8A	1
LRP8	1
CD1A	1
CD1B	1
CD1E	1
APOA2	1
PGLYRP3	1
UBASH3A	1
ICOSLG	1
FCN2	1
FCRL3	1
PGLYRP2	1
ALOX15	1
TREML1	1
IL23R	1
GFI1	1
DCST1	1
TRAT1	1
PYHIN1	1
CTLA4	1
CD200R1	1
OTOP1	1
RAET1E	1
PRKACG	1
MBL2	1
CLEC4D	1
NOD2	1

KIR3DL1	1
KLK5	1
KRT1	1
IL12A	1
BTNL3	1
IL13	1
GCSAML	1
APLF	1
FPR2	1
MUC7	1
IFNB1	1
MUCL1	1
THEMIS	1
GPR151	1
AC097637.1	1
TLR10	1
TLR6	1
GCSAM	1
LEP	1
NLRP6	1
NPPA	1
ZNF683	1
ATAD5	1
HMGB4	1
CD19	1
FUT7	1
MUC16	1
NLRP10	1
IFNL1	1
CLEC4G	1
IFNL2	1
COLEC10	1
MUC6	1
SFTPA2	1
FFAR3	1
BTLA	1

CARD9	1
C17orf99	1
TREML4	1
IFNA2	1
KIR2DL4	1
NCR1	1
PAX5	1
IFNL3	1
IL27	1
ELANE	1
CR1L	1
FCGR1B	1
CLEC4C	1
SH2D1B	1
RAET1G	1
BTNL2	1
CD300LD	1
NCR3	1
MUC21	1
MOG	1
MUC12	1
CLEC6A	1
SERPINB4	1
PVRIG	1
LAT	1
TREX1	1
KLRK1	1
GPR33	1
NLRP2B	1
MUC5AC	1
HMSD	1
PSMB11	1
USP17L2	1
LTA	1
TNF	1
IRGM	1

TLR9	1
LILRA4	1
LILRA2	1
KIR3DL2	1
TICAM2	1
KIR2DL3	1
CFHR1	1
CD8B2	1
INS	1
CLEC12B	1
AC136428.1	1
AL121845.2	1
AL121845.3	1
CD300LG	0.932205598
TGFB2	0.91398622
TLR5	0.912152494
CR1	0.903498472
FCRLB	0.879618132
NCR3LG1	0.867630697
CLNK	0.823461036
AMBP	0.780655814
MAP2K6	0.776490451
PRKCE	0.772405633
POLR3B	0.738650997
C7	0.732431688
ULBP3	0.724365545
SFTPD	0.714930736
SPHK2	0.710786046
NPY5R	0.670985016
PLA2G4A	0.655808995
GATA2	0.65553942
PRKCQ	0.649542074
CEACAM1	0.645508784
DENND1B	0.632168552
CD1C	0.620748805
FCER1A	0.594123743

PRKCZ	0.521413963
LRRC19	0.516177576
C5	0.503655266
FYB2	0.406810368
CR2	0.399156579
FOXJ1	0.387180613
VTCN1	0.350900998
PLPP4	0.347858956
APCS	0.311925002
TYRO3	0.288154866
KLK7	0.248747212
SLC22A13	0.232822302
S100A14	0.180999736
SUSD4	0.131147338
IL1RL1	0.121543365
GATA3	0.100845248
HRG	0.091490837
MUC15	0.035645108

Table S2. 63 PC genes and 365 NC genes

Gene name	Ensembl gene id
AKT1	ENSG00000142208
ARID1A	ENSG00000117713
BAP1	ENSG00000163930
BCL2	ENSG00000171791
BIRC5	ENSG00000089685
C5AR1	ENSG00000197405
CA9	ENSG00000107159
CAV1	ENSG00000105974
CCL2	ENSG00000108691
CD163	ENSG00000177575
CD24	ENSG00000272398
CD274	ENSG00000120217
CD4	ENSG00000010610
CD44	ENSG00000026508
CD68	ENSG00000129226
CDH1	ENSG00000039068
CDKN1A	ENSG00000124762
CDKN1B	ENSG00000111276
CDKN2A	ENSG00000147889
CIP2A	ENSG00000163507
CTNNB1	ENSG00000168036
CXCL9	ENSG00000138755
CXCR4	ENSG00000121966
DAG1	ENSG00000173402
DEFB1	ENSG00000164825
EDNRB	ENSG00000136160
EIF4EBP1	ENSG00000187840
EPAS1	ENSG00000116016
EPCAM	ENSG00000119888
EZH2	ENSG00000106462
FAS	ENSG00000026103
FOXM1	ENSG00000111206
FOXP3	ENSG00000049768

GALNT10	ENSG00000164574
HIF1A	ENSG00000100644
HPSE	ENSG00000173083
ICOS	ENSG00000163600
IGF1R	ENSG00000140443
IGF2BP3	ENSG00000136231
JAG1	ENSG00000101384
LAG3	ENSG00000089692
MET	ENSG00000105976
MUC1	ENSG00000185499
NOTCH1	ENSG00000148400
OTOF	ENSG00000115155
PBRM1	ENSG00000163939
PDGFRB	ENSG00000113721
PDZK1	ENSG00000174827
PLIN2	ENSG00000147872
PSMD9	ENSG00000110801
PTEN	ENSG00000171862
RGS5	ENSG00000143248
RPS6	ENSG00000137154
RXRA	ENSG00000186350
SERPINE1	ENSG00000106366
SETD2	ENSG00000181555
TGFBR2	ENSG00000163513
TP53	ENSG00000141510
VASH1	ENSG00000071246
VCAM1	ENSG00000162692
VHL	ENSG00000134086
VIM	ENSG00000026025
YAP1	ENSG00000137693

Table S3. Gene frequency order

Gene frequency order	Gene name	Frequency
**1**	IL4	4241
**2**	RNF135	3285
**3**	NLRC3	2931
**4**	NOD2	2926
**5**	HHLA2	2754
**6**	BAX	2636
**7**	EIF2B1	2593
**8**	KIR2DL4	2590
**9**	SRC	2172
**10**	IFNL1	2134
**11**	PDE4B	2049
**12**	GPS2	1901
**13**	HAVCR2	1885
**14**	OSCAR	1781
**15**	GRN	1761
**16**	POLR3G	1676
**17**	IL27RA	1637
**18**	BTNL3	1594
**19**	C1RL	1527
**20**	FCGR1A	1491
**21**	CD226	1468
**22**	TNFAIP3	1405
**23**	TEC	1402
**24**	B2M	1382
**25**	ARRB2	1380
**26**	KIR2DL1	1331
**27**	TRIM21	1309
**28**	XCL1	1291
**29**	IL18RAP	1285
**30**	P2RX7	1277
**31**	KCNN4	1276
**32**	GBP1	1264
**33**	CD79A	1248

**34**	DDX58	1242
**35**	HMOX1	1236
**36**	IGLL5	1220
**37**	CD84	1169
**38**	PVRIG	1163
**39**	C1QC	1145
**40**	IRAK3	1130
**41**	FCER2	1126
**42**	SAMHD1	1125
**43**	RGCC	1124
**44**	PRAM1	1107
**45**	ZBP1	1039
**46**	PLD2	1027
**47**	TESPA1	1024
**48**	FYN	1010
**49**	ZC3H12A	1010
**50**	SLAMF6	1001
**51**	NCF1	999
**52**	PRKD2	972
**53**	PSMB8	959
**54**	PTPN1	941
**55**	CARD11	934
**56**	CD160	934
**57**	IFI35	933
**58**	MAD2L2	922
**59**	APOBEC3F	921
**60**	MYO1G	911
**61**	ITGB2	908
**62**	CYBA	907
**63**	BIRC3	893
**64**	FFAR2	886
**65**	LIME1	868
**66**	C1S	866
**67**	WAS	864
**68**	TASL	857
**69**	SERPING1	852

**70**	PSMB4	845
**71**	APOE	837
**72**	BTN2A2	834
**73**	PLK2	829
**74**	HRAS	828
**75**	RARA	824
**76**	RFTN1	809
**77**	FPR2	805
**78**	NLRP3	805
**79**	TBX21	805
**80**	IL15	799
**81**	ENPP3	797
**82**	OAS1	789
**83**	LBP	782
**84**	CD79B	751
**85**	POLR3D	748
**86**	CACNB3	738
**87**	ADCY3	728
